# Supplementary material for: Allele-specific editing ameliorates dominant retinitis pigmentosa in a transgenic mouse model
Source: Am J Hum Genet. 2021 Jan 27;108(2):295–308. doi: 10.1016/j.ajhg.2021.01.006 (PMC7896132; doi:10.1016/j.ajhg.2021.01.006)
Supplement: Document S1. Figures S1–S9 [file mmc1.pdf]

The American Journal of Human Genetics, Volume *108*

Supplemental Data

Allele-specific editing ameliorates dominant retinitis pigmentosa in a transgenic mouse model

Clarissa Patrizi, Manel Llado, Daniela Benati, Carolina Iodice, Elena Marrocco, Rosellina Guarascio, Enrico M. Surace, Michael E. Cheetham, Alberto Auricchio, and Alessandra Recchia

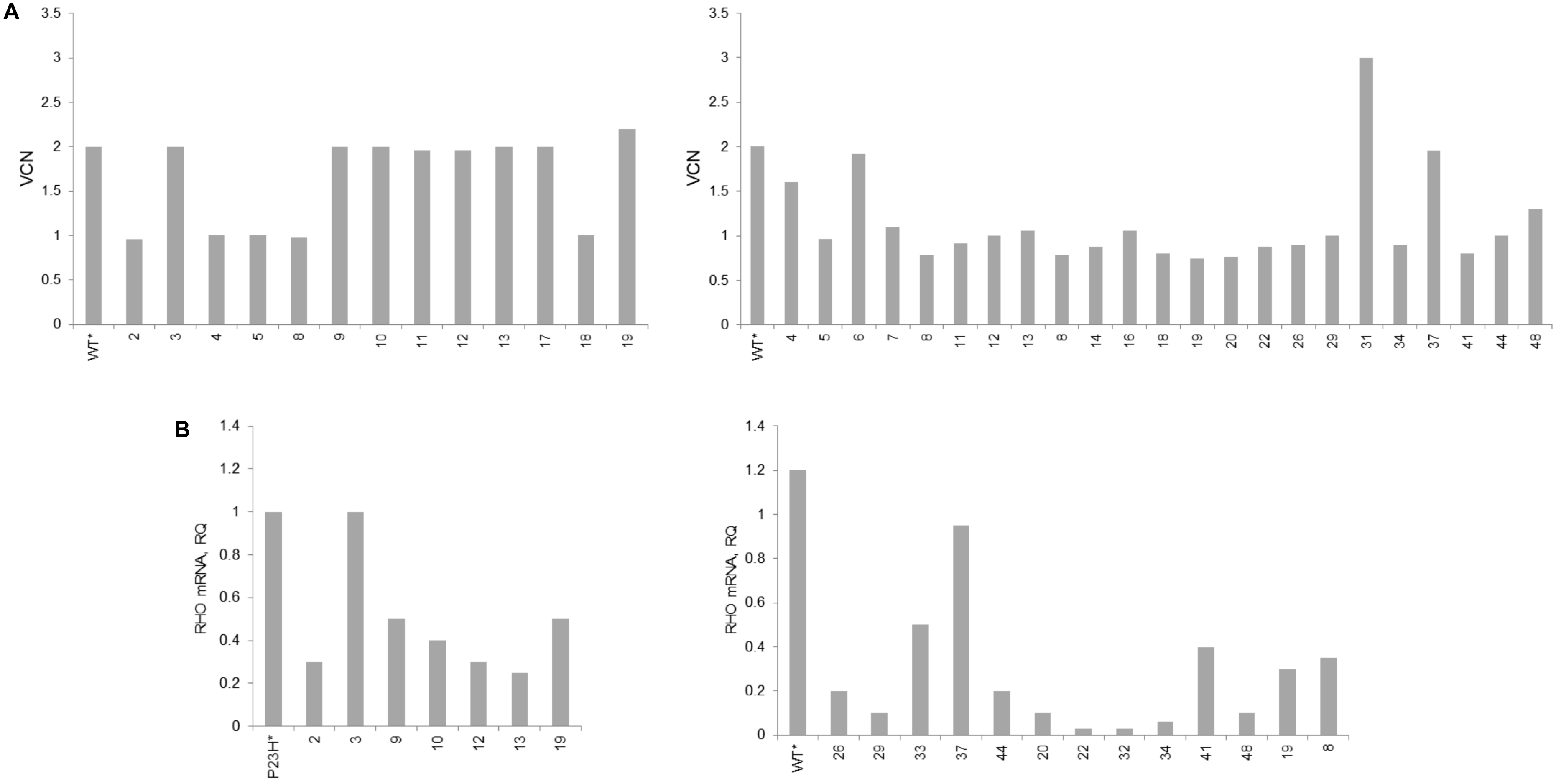

Figure S1. Engineering HeLa cells to express WT or P347S *RHO. A*) qPCR on P347S (left panel) and WT (right panel) *RHO* HeLa clones to determine vector copy number (VCN) of PGK-driven expression cassettes carrying WT or P347S *RHO* cDNA and part of the 3'UTR region. To select clones hosting 2 copies of the WT or P347S transgene, a WT* clone isolated and used in Latella *et al.*[29] was included as reference. **B)** Quantitative expression of *RHO* mRNA in P347S (left panel) or WT *RHO* (right panel) clones compared to a P23H* and WT* clones isolated and used in Latella *et al*.[29] . Clone #3 belonging to P347S RHO and clone #37 belonging to WT RHO were selected for *in vitro* editing experiments performed in this study.

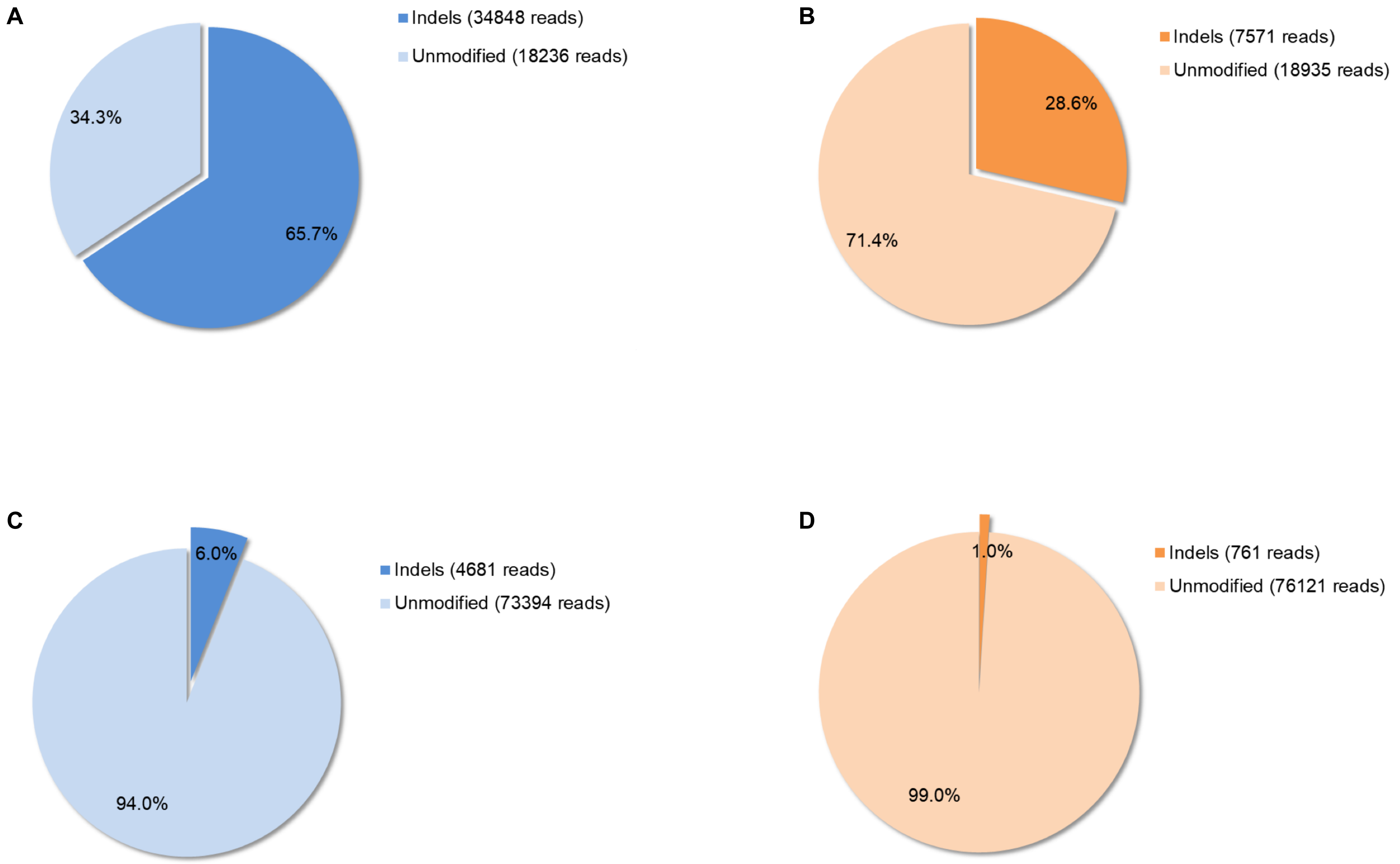

Figure S2. Allele-specific editing of P347S RHO transgene *in vitro*. P347S HeLa clone transfected with SpCas9_gRNA1 (**A**) or VQRHF1-SpCas9_gRNA5 (**B**) underwent NGS analysis tailored to the target site. NGS sequence reads were analyzed by CRISPResso software which retrieved a frequency of reads bearing indels or unmodified. Pie charts of the results from a representative experiment are reported. **C, D)** Pie charts of a representative experiment showing frequency and amount of reads carrying indels or unmodified reads scored in WT *RHO* HeLa clone transfected with SpCas9_gRNA1 (**C**) or -VQRHF1-SpCas9_gRNA5 (**D**).

A

	Potential splice site modified	Unmodified
THRA	0%	100%
ROR1	0%	100%
RBSN	0%	100%
EPSILS1	0%	100%

B

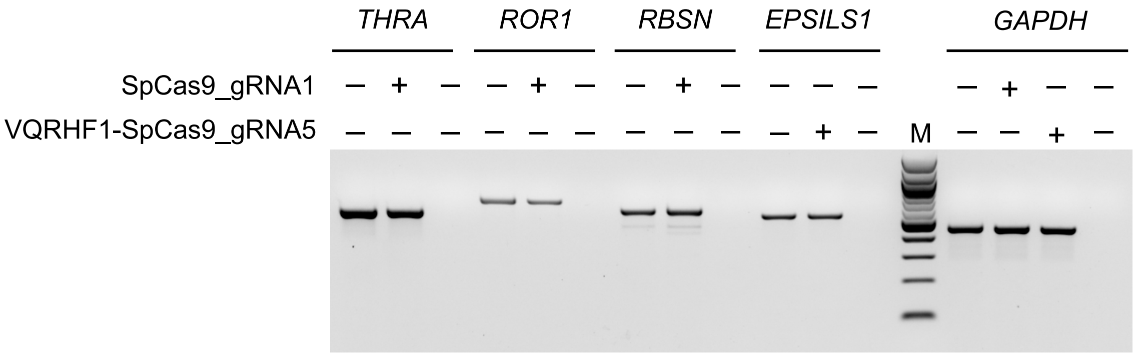

C

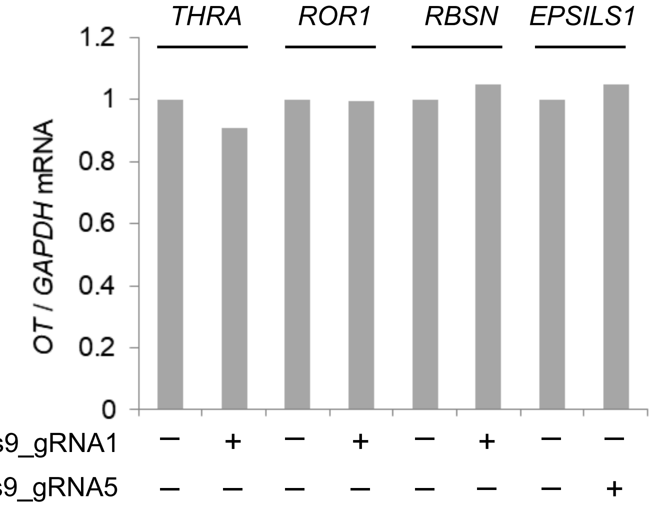

Figure S3. Unmodified expression of off-target genes upon CRISPR-mediated editing. A) CRISPResso analysis of potential splice sites in *THRA*, *ROR1*, *RBSN* and *EPSILS1* modified upon editing induced by SpCas9_gRNA1 or VQRHF1-SpCas9_gRNA5. **B)** RT-PCR analysis for the expression of *THRA*, *ROR1*, *RBSN* and *EPSILS1* in human RPE cells transfected or not with effector plasmids for the expression of SpCas9_gRNA1 or VQRHF1-SpCas9_gRNA5. RT-PCR analysis of *GAPDH* expression was evaluated as control. M, 100 bp molecular weight marker. **C)** Densitometric quantification of *THRA*, *ROR1*, *RBSN* and *EPSILS1* mRNA (OT), normalised to *GAPDH* in RPE cells transfected or not as indicated in panel (B).

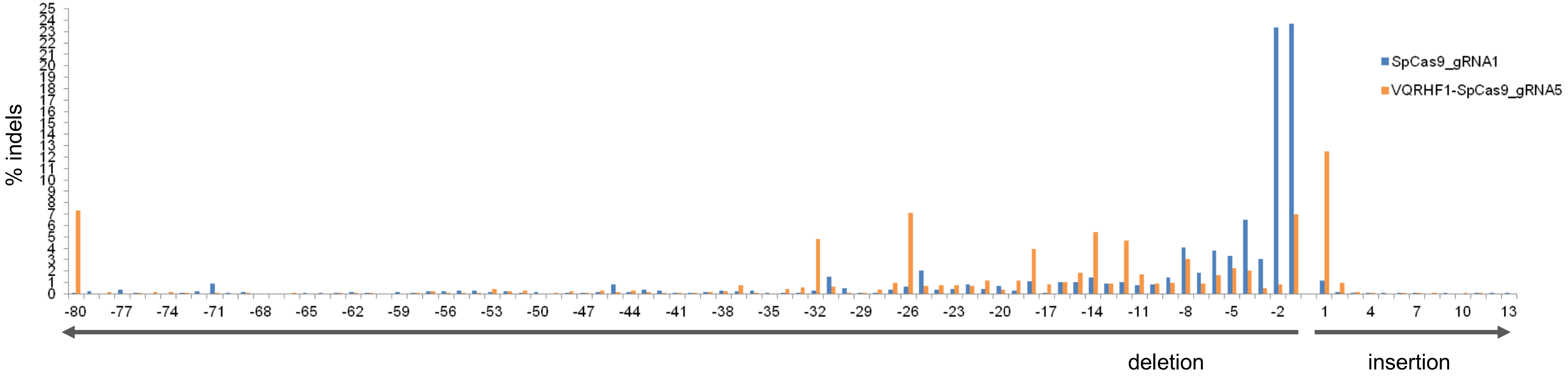

Figure S4. Distribution of indels in CRISPR-edited P347S *RHO* HeLa clone. Profile of indels scored in P347S *RHO* HeLa clone transfected with SpCas9_gRNA1 or VQRHF1-SpCas9_gRNA5. Deletions are represented by minus numbers whereas plus numbers represent insertions. Sequences without indels (value = 0) are omitted from the chart.

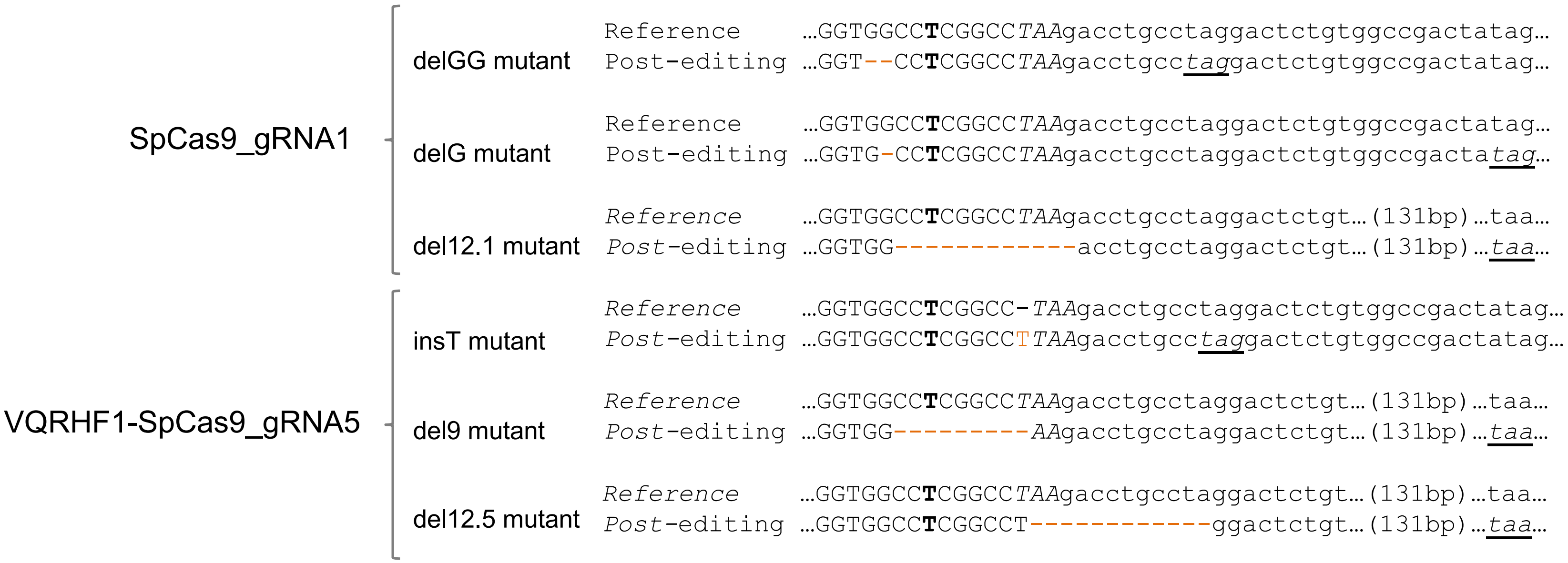

Figure S5. Most frequent RHO mutants generated upon CRISPR-mediated editing of P347S *RHO* mutation *in vitro*. Nucleotide sequence of the six most frequent mutants identified by CRISPResso upon editing mediated by SpCas9_gRNA1 or VQRHF1-SpCas9_gRNA5. The C to T conversion resulting in P347S mutation is highlighted in bold. Nucleotides belonging to the 3'-UTR are in italic. Dash indicates a deleted nucleotide, while insertion is shown in orange. New stop codons downstream the canonical one (capital letters in italics) are underlined.

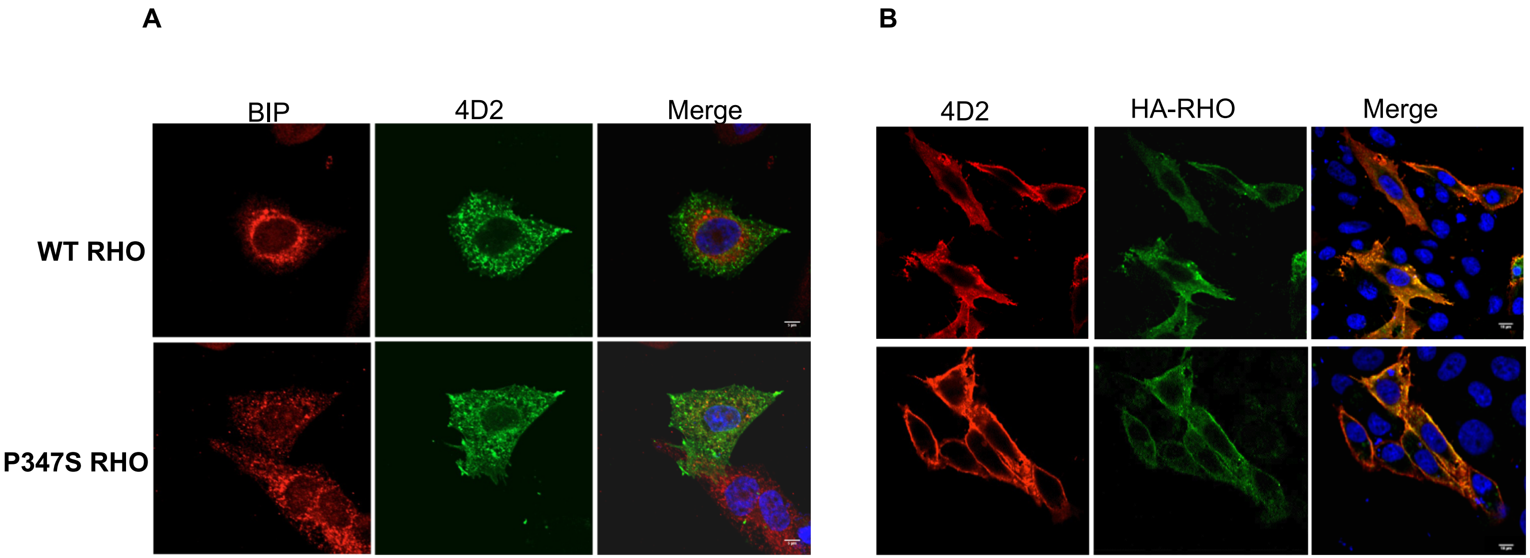

Figure S6. Localization of WT and P347S RHO protein in CHO cells. Immunofluorescence analyses of WT and P347S RHO protein performed in CHO cells transfected with plasmids expressing RHO fused at the N-terminal region to a human influenza hemagglutinin (HA) tag. **A**) Permeabilized cells were stained with anti-BIP and 4D2 antibodies and scale bar is 5 µm. **B**) Non-permeabilized cells were stained with 4D2 and anti-HA antibodies and scale bar is 10 µm. The panels generated merging the signals includes also a DAPI staining of the nuclei.

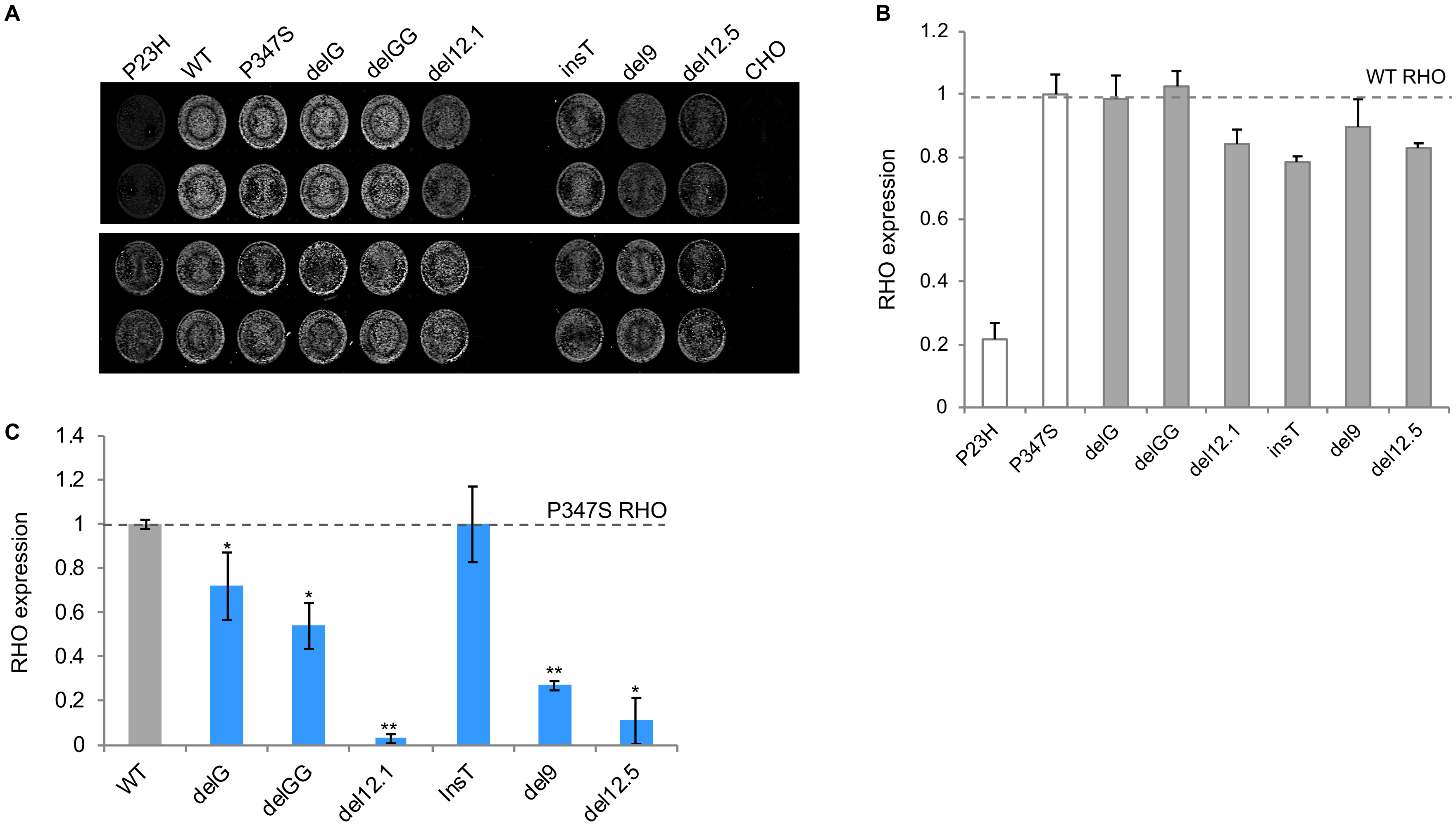

Figure S7. Localization and degradation of RHO mutants generated upon editing of P347S RHO mutation *in vitro*. **A)** In-Cell Western analysis of non-permeabilized (top panel) and permeabilized (bottom panel) CHO cells transfected with RHO mutant plasmids and stained with anti-HA antibody. As control P23H, WT and P347S RHO expression plasmids were transfected. Mock transfected cells (CHO) are included in the assay. **B)** Graphic representation of the ratio between rhodopsin localized to the plasma membrane over the total rhodopsin detected in "In Cell Western" analysis. The experiment was performed in duplicate and presented as mean ± SEM. **C)** Densitometric quantification of RHO protein level detected by western blot (Figure 2B) in CHO cells transfected with plasmids for the expression of RHO P347S, WT or mutants. The experiment was performed in duplicate and presented as mean ± SEM. * p-value < 0.05, ** p-value < 0.01.

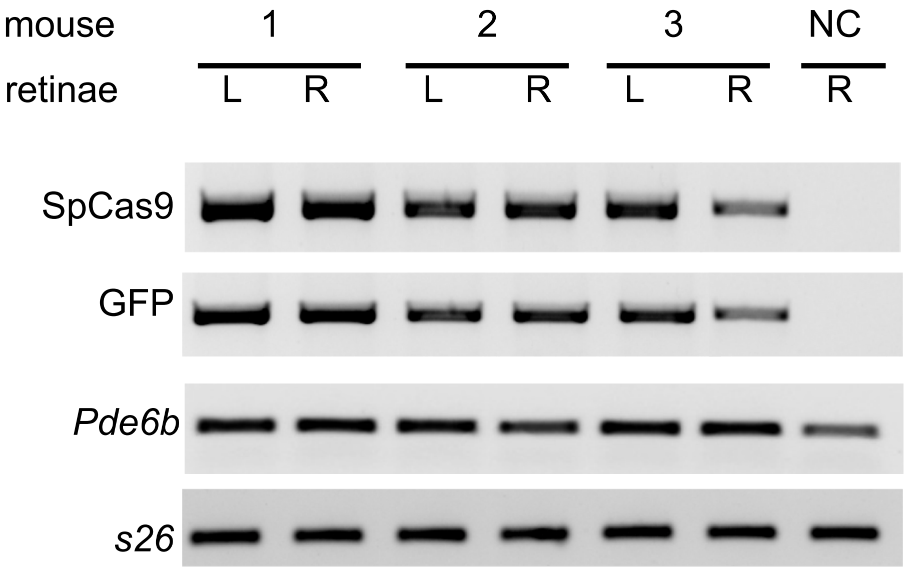

Figure S8. Expression of AAV-CRISPR effector vectors in P347S transgenic mice. RT-PCR analysis for the expression of SpCas9 (WT, mouse #1 and #2 L and R or VQRHF1, mouse #3 L and R) and GFP in retinae of P347S transgenic mice treated with effector or control AAV2/8 vectors. The expression of the rod photoreceptor gene *Pde6b* and the ribosomal *s26* RNA was used for normalization. L and R indicate left and right eye, respectively. NC, negative control, not injected retina.

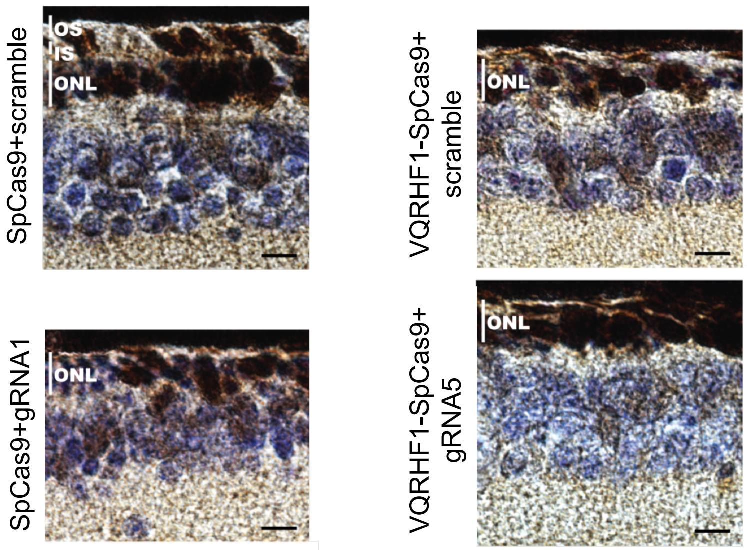

Figure S9. GFP expression in P347S transgenic mice treated with CRISPR-AAVs. Immunohistochemistry of retinal sections derived from mice injected with SpCas9+gRNA1, VQRHF1-SpCas9+gRNA5 or SpCas9+scramble vectors. Anti-GFP antibody stained the AAV carrying the gRNAs. Outer segment (OS), inner segment (IS) and outer nuclear layer (ONL) are indicated. Scale bar corresponding to 10 μm is shown.
