# Supplementary material for: Allele-specific editing ameliorates dominant retinitis pigmentosa in a transgenic mouse model
Source: Am J Hum Genet. 2021 Jan 27;108(2):295–308. doi: 10.1016/j.ajhg.2021.01.006 (PMC7896132; doi:10.1016/j.ajhg.2021.01.006)
Supplement: Document S2. Article plus supplemental information [file mmc5.pdf]

Allele-specific editing ameliorates dominant retinitis pigmentosa in a transgenic mouse model

Clarissa Patrizi,[1] Manel Llado,[2] Daniela Benati,[1] Carolina Iodice,[2] Elena Marrocco,[2] Rosellina Guarascio,[3] Enrico M. Surace,[2,5] Michael E. Cheetham,[3] Alberto Auricchio,[2,4,*] and Alessandra Recchia[1,*]

Summary

Retinitis pigmentosa (RP) is a group of progressive retinal degenerations of mostly monogenic inheritance, which cause blindness in about 1:3,500 individuals worldwide. Heterozygous variants in the rhodopsin (*RHO*) gene are the most common cause of autosomal dominant RP (adRP). Among these, missense variants at C-terminal proline 347, such as p.Pro347Ser, cause severe adRP recurrently in European affected individuals. Here, for the first time, we use CRISPR/Cas9 to selectively target the p.Pro347Ser variant while preserving the wild-type *RHO* allele *in vitro* and in a mouse model of adRP. Detailed *in vitro*, genomic, and biochemical characterization of the rhodopsin C-terminal editing demonstrates a safe downregulation of p.Pro347Ser expression leading to partial recovery of photoreceptor function in a transgenic mouse model treated with adeno-associated viral vectors. This study supports the safety and efficacy of CRISPR/Cas9-mediated allele-specific editing and paves the way for a permanent and precise correction of heterozygous variants in dominantly inherited retinal diseases.

Introduction

Retinitis pigmentosa (RP) is a group of genetically heterogeneous retinal diseases afflicting three million people across the globe with an incidence of 1 in 3,500 live births.[1–4] Individuals affected by RP initially manifest night-blindness with gradual constriction of the visual field but sparing of central vision. As the rod loss progresses, secondary death of cones occurs, leading to deterioration of visual acuity and eventual blindness. Part of the difficulty in treating RP is its complex and diverse genetic etiology. To date, more than 3,000 disease-associated variants in approximately 70 disease-causing genes have been causally associated with RP[5] and currently more than 150 documented missense/nonsense variants in the rhodopsin (*RHO*) gene are associated with an autosomal dominant RP (adRP) phenotype (RP4 [MIM: 613731]).[6,7] The *RHO* gene encodes for rhodopsin (RHO), a visual pigment found in rod photoreceptors, responsible for converting photons into chemical signals initiating vision. Rhodopsin is a 348 amino acid G-protein-coupled receptor characterized by an extracellular N-terminal domain needed to stabilize the protein, seven transmembrane-spanning α helices hosting the binding site for the chromophore 11-cis-retinal, and an intracellular C-terminal domain, involved in vectorial transport of rhodopsin to rod outer segments (OSs).[8–11] Although approximately half of the RHO-associated adRP cases in the US are caused by the substitution of proline to histidine at position 23 (p.Pro23His)[12] in the extracellular N-terminal domain, class I variants clustered in the C-terminal domain[6] give rise to a defect in post-Golgi trafficking to the OS and result in a more severe phenotype and worse prognosis for affected individuals.[13–15] The large majority of *RHO* pathogenic variants are inherited in an autosomal dominant manner. In most of these cases, simply adding a normal copy of the gene is not sufficient,[16] as the affected gene needs to be inactivated. Downregulation of RHO variants has been attempted in disease models using ribozymes[17], RNA interference,[16,18] and transcriptional repressor by zinc finger proteins.[19,20] Most of these approaches do not distinguish the disease-associated alleles from the wild-type (WT), thus achieving bi-allelic suppression that also requires addition of a WT *RHO* cDNA (“suppression and replacement”). The ability to correct disease-causing variants while sparing the WT allele has been improved greatly by the discovery of CRISPR/Cas9 genome editing.[21] Cas9 endonucleases generate double-strand breaks (DSBs) in a specific genomic region that is located adjacent to a protospacer-adjacent motif (PAM) and targeted by a complementary guide RNA (gRNA).[22] In the absence of the exogenous template, the Cas9-induced DSBs are repaired through the non-homologous-end-joining (NHEJ) mechanism, leading to the frequent introduction of insertions or deletions in the target site. Thereby, as a valid alternative to the “suppression and replacement” approach that may be potentially used to treat a wide array of dominant diseases but that requires a double intervention, specific inactivation of the altered allele can be pursued for dominant-negative and gain-of-function variants[23] that generate a unique PAM site or allow the design of a gRNA that contains the variant in the seed sequence.

[1]Centre for Regenerative Medicine, Department of Life Sciences, University of Modena and Reggio Emilia, 41125 Modena, Italy; [2]Telethon Institute of Genetics and Medicine, 80078 Pozzuoli, Italy; [3]UCL Institute of Ophthalmology, London EC1V 9EL, UK; [4]Medical Genetics, Department of Advanced Biomedicine, Federico II University, 80125 Naples, Italy; [5]Medical Genetics, Department of Translational Medicine, Federico II University, 80125 Naples, Italy

*Correspondence: alessandra.recchia@unimore.it (A.R.), auricchio@tigem.it (A.A.)
https://doi.org/10.1016/j.ajhg.2021.01.006.
© 2021 The Authors. This is an open access article under the CC BY license (http://creativecommons.org/licenses/by/4.0/).

Given the high prevalence of the c.68C>A *RHO* allele encoding the p.Pro23His variant in the United States,[24] it is not surprising that this has been the primary target of CRISPR/Cas9-mediated gene editing. Indeed, this strategy has already been demonstrated to be effective in recent studies employing the Pro23His knockin mouse model.[12,21] In these reports, the authors showed a reduced expression of the disease-associated murine transcript triggered by NHEJ repair occurring in the first exon of the gene. The allele-specific inactivation of the murine allele encoding the p.Pro23His variant resulted in a delay of the degenerative retinal process and rescue of retinal functional activity.

A gene editing approach tailored to the C-terminal domain of human rhodopsin and, in particular, to proline 347, the most common residue affected in European individuals,[25] has been neglected so far. Here, for the first time, we employ both *Streptococcus pyogenes* Cas9 (SpCas9) WT and the high-fidelity variant carrying seven amino acid substitutions, Asn497Ala, Arg661Ala, Gln695Ala, Gln926Ala, Asp1135Val, Arg1335Gln, and Thr1337Arg (hereafter referred to as the VQRHF1),[26,27] combined with allele-specific gRNAs to edit the c.1039C>T variant in *RHO,* which leads to the p.Pro347Sser RHO variant. We characterize in detail c.1039C>T *RHO* allele-specific editing and the predicted genome-wide off-target sites by next-generation sequencing (NGS). Considering the role of the RHO C terminus in protein trafficking/folding and the unpredictable editing occurring at the target site, we have performed in-depth biochemical analyses of the most frequent RHO variants generated upon CRISPR/Cas9-mediated editing. Moreover, subretinal delivery of adeno-associated virus (AAV) vector serotype 2/8 (AAV2/8) carrying the WT or VQRHF1 SpCas9 and the target or scramble gRNA demonstrates the therapeutic potential of AAV-Cas9 gene editing to inactivate the human p.Pro347Ser pathogenic variant in the transgenic Pro347Ser mouse model, ameliorating disease progression.

Material and methods

Plasmids

To generate the pCCL.PGK.wtRHO+3′UTR, a 250 bp region of the RHO 3′ UTR was amplified from the genomic DNA of Pro23His transgenic mice[28] and cloned into pCCL-PGK.wtRHO[29] downstream the stop codon of the WT RHO cDNA. To generate the pCCL.P347S.RHO+3′UTR, the region including the exon 5 of *RHO* cDNA, carrying the p.Pro347Ser variant, and a 250 bp region of the *RHO* 3′ UTR amplified from the genomic DNA of Pro347Ser transgenic mouse[14] were cloned into the pCCL-PGK.wtRHO backbone downstream of the exon 4 of *RHO* cDNA. The effector plasmid SpCas9_gRNA1 was generated by cloning the gRNA1 in the pX330-U6-Chimeric_BB-CBh-hSpCas9 plasmid (Addgene: 42230) by oligo annealing into *BbsI* sites. The effector plasmid VQRHF1-SpCas9_gRNA5 was obtained by cloning the gRNA5 in the pX330-U6-Chimeric_BB-CBh-hSpCas9 plasmid by oligo annealing into *BbsI* sites, followed by subcloning of the U6-gRNA5 cassette into the MSP2440 plasmid (Addgene: 72250) expressing the VQRHF1-SpCas9.[27] To generate effector plasmids carrying Hygromycin resistance gene, the expression cassette for the Hygromycin resistance gene under the control of herpes simplex virus thymidine kinase (TK) promoter was subcloned in SpCas9_gRNA1 and VQRHF1-SpCas9_gRNA5 plasmids downstream of the polyA signal of SpCas9 expression cassette, generating SpCas9_gRNA1-TKHygro and VQRHF1-SpCas9_gRNA5-TKHygro. To generate CMV.HA.wtRHO+3′UTR and CMV.HA.P347SRHO+3′UTR plasmids expressing RHO under the cytomegalovirus (CMV) promoter, the h*RHO* cDNA carrying the WT or p.Pro347Ser variant and the 250 bp region of *RHO* 3′ UTR were cloned into CMV.HA.RHO plasmid.[30] RHO variant plasmids (CMV.HA.RHO delG/delGG/del12.1/InsT/del9/del12.5) were generated by using the Site-Directed Mutagenesis Kit (SDM) (NEB, New England Biolabs, Ipswich, MA, USA) according to manufacturer's protocol. Primer pairs (Table S3) were designed for the incorporation of insertions or deletions into CMV.HA.P347S.RHO+3′UTR and pCCL.PGK.P347S.RHO+3′UTR plasmids. The pAAV2.1-U6-gRNA1-RHO-GFP, pAAV2.U6-gRNA5.RHO-GFP, and pAAV2.1-U6-scramble-RHO-GFP plasmids were generated by cloning the expression cassette for gRNA1, gRNA5, or scramble gRNA into a pAAV2.1-RHO-GFP plasmid[31] upstream of the RHO promoter by *AflII* restriction. The pAAV2.1-IRBP-SpCas9-spA plasmid was generated by cloning the interphotoreceptor retinol binding protein (IRBP) promoter into pAAV-pMecp2-SpCas9-spA (Addgene: PX551) using HindIII and *AgeI* restriction enzymes. The pAAV2.IRBP.VQR-HF1.SpCas9 vector was generated by cloning the CMV.VQR-HF1.SpCas9-BGHpA cassette into the AAV2.1 backbone[32] followed by replacing the CMV promoter with IRBP.

Cell culture

HeLa, CHO, HEK293T, and hTERT-RPE cells were obtained from the American Type Culture Collection (ATCC) and were cultured in Dulbecco's modified Eagle's medium (DMEM) supplemented with 10% fetal calf serum (FCS), 100 U/mL penicillin, and 100 mg/mL streptomycin (Lonza, Basel, Switzerland). For protein degradation assay, CHO cells transfected with CMV-HA-Pro347Ser, WT, and variant RHO plasmids were treated with 10 μg/mL of cycloheximide (CHX) or MG-132 (50 μM) and analyzed after0, 3, and 6 h or 0, 4, and 6 h, respectively.

Viral production

Lentiviral vectors (LVs) pseudotyped with the vesicular stomatitis virus G protein were prepared by transient co-transfection of HEK293T cells with transfer vector, pMD.Lg/pRRE.Int, pMD2.VSV-G envelope-encoding packaging plasmid, and pRSV-Rev.[29] AAV vectors were produced by triple transfection of HEK293 cells followed by two rounds of $CsCl_2$ purification.[32] For each viral preparation, physical titers (GC/mL) were determined by averaging the titer achieved by dot-blot analysis[33] and by PCR quantification using TaqMan (Applied Biosystems, Carlsbad, CA, USA). The probes used for dot-blot and PCR analyses were designed to anneal with the IRBP promoter for the pAAV2.1-IRBPSpCas9-spA vector and the bGHpA region for vectors encoding for gRNA expression cassettes and RHO-GFP. The length of probes varied between 200 and 700 bp.

Transfections of cells, isolation of single-cell clones, and vector copy number determination

Transfection of 2.5 × 10^5 HeLa cells was obtained using Fugene HD transfection reagent (Promega, Madison, WI, USA) following the manufacturer's instructions. For each transfection reaction, 2 μg of plasmid DNA were mixed to 6 μL Fugene (3:1 ratio).

Transfection of 2.5×10^5 HEK293T cells was performed using $CaPO_4$ protocol with 1 μg of SpCas9_gRNA1-TKHygro or VQRHF1-SpCas9_gRNA5-TKHygro. Starting from the day after transfection, cells were treated with 0.2 mg/mL of Hygromycin for 15 days to select antibiotic resistant cells.

Transfection of 1×10^5 hTERT-RPE cells was performed with 1 μg of SpCas9_gRNA1 or VQRHF1-SpCas9_gRNA5 or respective control plasmids using TransIT-XI (Mirus Bio, Madison, WI, USA) following the manufacturer's instructions. Transfection of 2.5×10^4 CHO cells was obtained using Transit-XI following the protocol instructions. Each transfection reaction contained 150 ng of plasmid coding for p.Pro347Ser RHO or wt RHO or the selected RHO variants obtained upon gene editing in Pro347Ser RHO HeLa clone. To obtain HeLa clones expressing WT or p.Pro347Ser RHO, HeLa cells were transduced with LVs carrying the WT or p.Pro347Ser RHO expression cassettes. Transduced bulks were limiting diluted to obtain a concentration of 0.3 cells/well and seeded in a 96-well plate. Genomic DNAs (gDNAs) were extracted from single cell clones, and a PCR on the RHO expression cassette was performed as follows: primers PGK_F and hRHO_ex1_R (Table S3); PCR conditions, 30 s at 94°C, 30 s at 58°C, and 30 s at 72°C for 30 cycles. PCR products were separated on 1% TBE (Tris/Borate/EDTA)-agarose gels and stained with ethidium bromide for analysis.

For vector copy number (VCN) determination, qPCR was conducted with 20 ng gDNA using TaqMan Universal PCR Master Mix (Applied Biosystem) and probes specific for human *RHO* and glyceraldehyde 3-phosphate dehydrogenase (*GAPDH*) (*hRHO*, Hs00892431m1; *hGAPDH*, Hs03929097_g1; Applied Biosystems, Milan, Italy). Reactions were performed at 50°C for 2 min and 95°C for 10 min, followed by 40 cycles at 95°C for 15 s and 60°C for 1 min. Normalization to *GAPDH* in the same gDNA was performed and the relative copy number was calculated by using the $2-^{\Delta\Delta CT}$ quantification.

Semiquantitative and quantitative RT-PCR analyses

Total RNA from WT or Pro347Ser RHO HeLa clones, mice retinae, and hTERT-RPE cells[34] was isolated with the RNeasy Mini and Micro kits (QIAGEN, Hilden, Germany) according to the manufacturer's instructions. cDNA was synthesized in a 20 μL reaction by the Superscript III Reverse Transcription kit (Invitrogen). Semiquantitative RT-PCR analysis was performed with the following oligonucleotides: hRHO-Cterm_F and WPRE_R, GAPDH.F and GAPDH.R for mRNA analysis of HeLa cells, hRHO-Cterm_F and hRHO_3-′UTR_RC, Cas9.F and Cas9.R GFP.F and GFP.R, m.s26rRNA.F and m.s26rRNA.R, and PDE6b.F and PDE6b.R for mRNA analysis of treated mice retinae. PCR cycles were as follows: 94°C for 30 s, 58°C for 30 s, and 72°C for 30 s. TaqMam real-time PCR analysis was performed using the ABI Prism 7900 Sequence Detection System (Applied Biosystems, Monza, Italy) with TaqMan Universal PCR Master Mix and probes specific for human *RHO*, human and mouse *GAPDH*, and mouse *Pde6g* (*hRHO*, Hs00892431m1; *hGAPDH*, NM_02046.3; *mGAPDH*, Mm99999915_g1; *mPde6g*, Mm00501964_m1; Applied Biosystems, Monza, Italy). Reactions were performed at 50°C for 2 min and 95°C for 10 min, followed by 40 cycles at 95°C for 15 s and 60°C for 1 min. The relative expression of the target genes was normalized to the level of *GAPDH* housekeeping gene for HeLa clones or *Pde6g* photoreceptor housekeeping gene in the same cDNA by using the $2-^{\Delta\Delta CT}$ quantification. The replicated relative quantity (RQ) values for each biological sample were averaged.

Targeted deep sequencing and off-target analysis

Genomic DNA was extracted from HeLa clones transfected with SpCas9_gRNA1 or VQRHF1-SpCas9_gRNA5, HEK293T cells transfected with SpCas9_gRNA1-TKHygro or VQRHF1-SpCas9_gRNA5-TKHygro and selected with Hygromycin, and mice retinae using QIAamp DNA Mini or Micro kits (QIAGEN) following the manufacturer's instructions. For NGS analysis, the genomic regions flanking gRNA target sites were amplified by PCR using the AccuPrime Taq DNA Polymerase System (Thermo Fisher) and primers in Table S3. PCR products were subjected to library preparations. Briefly, primers hRHO-Cterm_F and WPRE_R and primers hRHO-Cterm_F and hRHO_3′UTR_RC (primers 1st PCR amplification) were, respectively, used to specifically amplify the p.Pro347Ser-coding *RHO* cDNA in HeLa stable clones and human p.Pro347Ser-coding *RHO* gene in transgenic mice. A second amplification with primers (primers 2nd PCR amplification**)** was required. For off-target analysis in Hygromycin-selected HEK293T cells, individual single pairs of primers were used (primers 1st PCR amplification). For NGS library preparation, individual barcode was added to each DNA fragment by a limited number (n = 8) of PCR cycles using primers detailed as primers 3rd PCR amplification. Equimolar amounts of library were mixed, diluted, and sequenced with an Illumina MiSeq system by CIBIO Trento. The percentage of indels was quantified by the CRISPResso webtool.

The off-target analysis for each gRNA was performed by using the COSMID webtool.[35] Off-targets analysis was performed by NGS and the percentage of indels was quantified by CRISPRessoV2 webtool.

Immunoblotting analysis

Cell lysates were extracted with RIPA buffer: 50 mM Tris-HCl pH 8.0, 150 mM w/v NaCl, 1 mM EDTA, 1% v/v NP-40, 0.1% w/v SDS, and 0.05% w/v sodium deoxycholate in the presence of 2% of protease inhibitor cocktail (Roche, Basilea, Switzerland).[36] Sixty μg of protein extracts from HeLa clones and 20 μg of protein extracts from transfected CHO cells were loaded on 12% sodium dodecyl sulfate (SDS)-polyacrylamide gel electrophoresis (PAGE). After electrophoresis, samples were transferred PVDF membranes (GE Healthcare). The membranes were incubated with monoclonal 4D2 primary antibody (1:500, Millipore, Burlington, MA, USA) or monoclonal anti-HA primary antibody (Sigma, 1:1,000) and anti-B-actin antibody (Abcam, Cambridge, UK) for protein loading normalization. Horseradish-peroxidase-conjugated anti-mouse antibody (diluted 1:10,000 for RHO variant expression analysis, 1:5,000 for rhodopsin expression in HeLa clones) was used for chemiluminescent detection (Pierce). Quantification was performed by densitometry analysis of scanned images using ImageJ software.

Immunofluorescence and "In-Cell Western" analysis

For immunofluorescence, cells were fixed using 4% v/v paraformaldehyde (PFA) and permeabilized with 0.01% v/v Triton X-100/PBS, while non-permeabilized cells remained in PBS. Non-specific binding sites were blocked using blocking solution consisting of 3% of bovine serum albumin (BSA) and 10% of normal goat serum in PBS. Non-permeabilized cells were incubated with 4D2 primary antibody (1:500, Millipore) and anti-HA primary antibody (1:1,000, Sigma) in blocking solution; permeabilized cells were incubated with anti-HA primary antibody (1:500, Sigma) and binding immunoglobulin protein (BIP) (1:200, Sigma). As secondary antibodies, Alexafluor488-conjugated goat

anti-Mouse (1:1,000) and Alexafluor594-conjugated goat anti-Rabbit (1:1,000) were used. After washing, incubation with 6-diamidino-2-phenylindole DAPI (1:5,000) was performed, then slides were mounted with Dako Fluorescent Mounting Medium (Dako Omnis). Immunofluorescence were visualized using Zeiss LSM 700 laser scanning confocal microscope and analyzed with ZEISS ZEN Microscope software.[37]

To perform "In-Cell Western" analysis, cells were fixed, treated with blocking solution, and stained with anti-HA antibody (1:1,000, Sigma). The secondary antibody IRDye 680RD goat anti-mouse (LI-COR Biosciences, Lincoln, NE, USA) was used. Acquisition was performed using the Odyssey Imager (LI-COR Biosciences).

Cytotoxicity

The Cytotoxicity Detection Kit (LDH, Roche) assay was used for the quantification of cell death based on the measurement of the lactate dehydrogenase (LDH) released from the cytosol of damaged cells into the supernatant. Transfected WT and Pro347Ser RHO HeLa clones were incubated with reaction mixture provided by the kit and prepared following the manufacturer's instructions. Not-transfected HeLa cells were used as "low control" (spontaneous LDH release), while Triton 100× was added to "high control" samples (maximum LDH release). The absorbance was measured at 492 nm by a spectrophotometer (Safire, Tecan, UK). The percentage of sample cytotoxicity was measured following this equation: cytotoxicity (%) = (exp. value − low control)/(high control − low control) × 100.

Animal care

Mice were housed at the TIGEM animal house (Pozzuoli, Italy) and maintained under a 12 h light/dark cycle. Pro347Ser transgenic mice were maintained as F0 by crossing them with themselves and were crossed with C57BL/6J mice purchased from Envigo Italy SRL (Udine, Italy) to generate experimental F1 mice.

Subretinal injection of AAV vectors in Pro347Ser transgenic mice

This study was carried out in accordance with the Association for Research in Vision and Ophthalmology Statement for the Use of Animals in Ophthalmic and Vision Research and with the Italian Ministry of Health regulation for animal procedures (Ministry of Health authorization number 147/2015-PR). Surgery was performed under general anesthesia, and all efforts were made to minimize animal suffering. One-week-old mice were anesthetized with an intraperitoneal injection of 2 mL/100 g of body weight of ketamine/medetomidine, then AAV2/8 vectors were delivered subretinally via a trans-scleral trans-choroidal approach, as described by Liang et al.[38] Eyes were injected with 1 μL of vector solution. The AAV2/8 dose (GC/eye) was 1×10^9 of each vector/eye, and thus, co-injection resulted in a maximum of 2×10^9 GC/eye.

Electrophysiological recordings and pupillary light response analysis

The retinal electrophysiological recordings of Pro347Ser mice were performed as previously described.[39] Pupillary light responses (PLRs) from Pro347Ser mice were recorded in dark condition using the TRC-50IX retinal camera connected to a charge-coupled device NikonD1H digital camera (Topcon Biomedical Systems). Mice were exposed to light stimuli at 1 lux for approximately 10 s and one picture per eye was acquired using the IMAGEnet software (Topcon Biomedical Systems). For each eye, the pupil diameter was normalized to the eye diameter (from temporal to nasal side).

Histological analysis

Mice were sacrificed and eyes were fixed in Davidson's fixative (deionized water, 10% acetic acid, 20% formalin, 35% ethanol) overnight, followed by dehydration in serial ethanols and embedding in paraffin blocks. Ten-μm thick microsections were cut along the horizontal meridian, progressively distributed on slides.

Paraffin-embedded mouse retinae were treated with a citrate buffer for antigen retrieval, incubated with primary antibodies, and developed with 3,3′-Diaminobenzidine (DAB) using a Bond-III Automated IHC Stainer from Leica Biosystems according to the manufacturer's instructions. The primary antibodies used were as follows: monoclonal 1D4 (1:1,000, a gift from Robert Molday), monoclonal 4D2 (1:30,000, Millipore, Burlington, MA, USA), and monoclonal anti-GFP (1:2,000, Proteintech, Manchester, UK). Images were acquired in bright field using a Zeiss LSM 510 AxioCam microscope.

Statistical analysis

Data were analyzed for statistical significance using two-way ANOVA or Student's t test. All values in each group were expressed as the mean ± SEM. All group comparisons were considered significant at $p < 0.05$, $p < 0.01$, and $p < 0.001$.

Results

CRISPR/Cas9 system specifically edits, *in vitro*, c.1039C>T *RHO* encoding the p.Pro347Ser variant

CRISPR/Cas9-mediated inactivation of dominant variants in *RHO* would in turn minimize and delay photoreceptor degeneration and visual loss in individuals affected by RP. To specifically target the p.Pro347Ser variant, which is caused by a C-to-T transition in *RHO* exon 5 (c.1039C>T), two gRNAs were designed. Guide RNA1 carries the variant (T) in the seed sequence, as the last nucleotide of the 20 nt protospacer, and guides the SpCas9 to the 5′-CGG-3′ PAM sequence. On the reverse complementary strand, gRNA5 guides the high-fidelity VQRHF1SpCas9 variant to the 5′-CGAG-3′ PAM sequence that includes the variant (A, in the reverse complementary strand) (Figure 1A).

In the absence of human cell lines constitutively expressing RHO, HeLa cells were engineered with a lentivirus expressing from the phosphoglycerate kinase (PGK) promoter, the WT, or p.Pro347Ser-coding *RHO* cDNA followed by a 250 bp-long region of the 3′ UTR for a more comprehensive analysis of the translated alternatives upon CRISPR-mediated editing. Two clones carrying two copies of WT or p.Pro347Ser-coding *RHO* cDNA (Figure S1A) that expressed RHO at comparable levels (Figure S1B) were selected and used for further experiments. To assess gRNA specificity and efficiency, WT and Pro347Ser RHO HeLa clones were transfected with effector plasmids expressing the gRNA1 or gRNA5 and the appropriate SpCas9 nuclease (SpCas9_gRNA1 or VQRHF1-SpCas9_gRNA5, respectively), or cognate plasmids without gRNAs as negative controls, and analyzed by NGS.

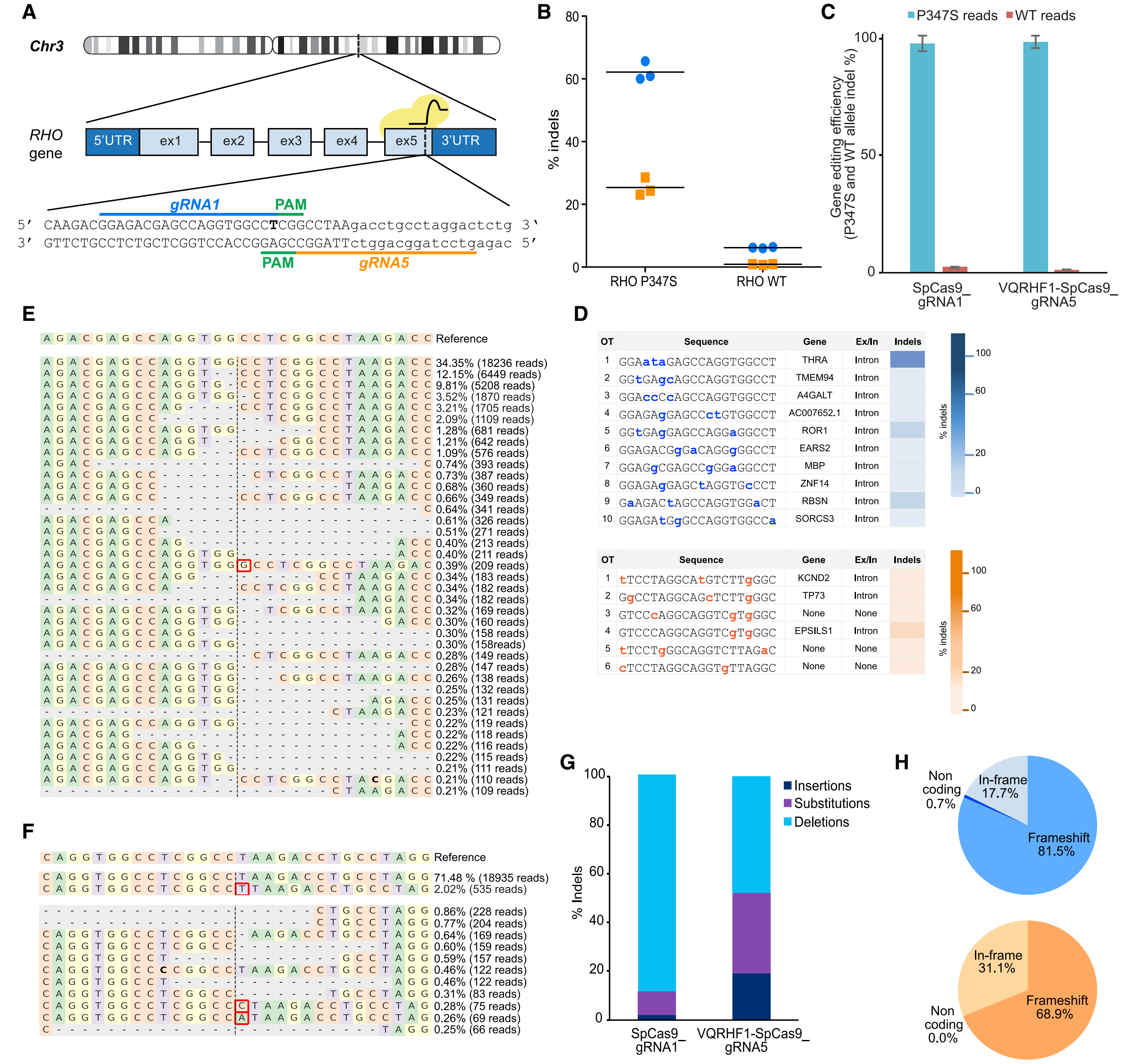

Figure 1. CRISPR/Cas9 targeting of c.1039C>T *RHO* dominant variant encoding p.Pro347Ser

(A) Schematic representation of human chromosome 3. The picture illustrates two gRNAs (gRNA1 and gRNA5) targeting the variant (T, in bold) in the exon 5 of *RHO* and the PAM sequences. Capital letters indicate the exon 5, whereas the 3′ UTR is in lowercase.

(B) CRISPResso analysis of NGS data obtained on Pro347Ser and WT RHO HeLa clones transfected with effector plasmids (SpCas9_gRNA1 in blue and VQRHF1-SpCas9_gRNA5 in orange). The experiment was performed in triplicate, and the mean is presented.

(C) CRISPResso analysis of indels occurring in c.1039C>T *RHO* transgene and in the endogenous WT *RHO* gene after transfection of effector plasmids in Pro347Ser RHO HeLa clone. The experiment was performed in triplicate and is presented as mean ± SEM.

(D) Indels analysis of off-target sites predicted for gRNA1 (top) and for gRNA5 (bottom). The color bars (blue and orange) represent values ascending from bottom to top ranking the indels frequency.

(E and F) CRISPResso graphic representation of indels scored in the target site of Pro347Ser RHO HeLa cells transfected with SpCas9_gRNA1 (E) and VQRHF1-SpCas9_gRNA5 (F). The top sequence is the unmodified reference. The percentage of indel frequency and the number of reads scored are indicated. Red boxes indicate nucleotide insertion and nucleotide in bold indicates substitution.

(G) Type of indels, and their relative percentage, generated in Pro347Ser RHO HeLa clone transfected with SpCas9_gRNA1 and VQRHF1-SpCas9_gRNA5.

(H) CRISPResso analysis of indels generated by SpCas9_gRNA1 (top pie chart) and VQRHF1-SpCas9_gRNA5 (bottom pie chart) leading to frameshift or in-frame alterations.

CRISPResso analysis[40] on sequence reads from the Pro347Ser RHO HeLa clone transfected with SpCas9_gRNA1 or VQRHF1-SpCas9_gRNA5 scored 65.7% and 28.6% editing, respectively, in a representative experiment (Figures S2A and S2B), while 6% and 1% of reads from transfected WT RHO clone were edited by gRNA1 and gRNA5, respectively (Figures S2C and S2D). NGS analysis on three independent experiments confirmed the efficiency and specificity of gRNA1 (62.0 ± 1.8) and gRNA5 (25.4% ± 1.6) in targeting the c.1039C>T *RHO* variant, with barely detectable editing of WT *RHO* (gRNA1, 6.2 ± 0.1; gRNA5, 0.9 ± 0.1) (Figure 1B). To further eliminate any bias due to different transfection efficiency between WT and Pro347Ser RHO HeLa clones, allele-specific editing generated by SpCas9_gRNA1 and VQRHF1-SpCas9_gRNA5 was analyzed in the Pro347Ser RHO HeLa clone by NGS targeted to the p.Pro347Ser-coding transgene copies or the endogenous WT *RHO* alleles. Allele-specific analysis showed that 97.7% and 98.5% of all indels generated in Pro347Ser RHO HeLa clone by SpCas9_gRNA1 or VQRHF1-SpCas9_gRNA5, respectively, occurred in the variant transgene (Figure 1C), confirming the highly specific editing of the c.1039C>T *RHO* variant. While the genomic landscape of the *RHO* region in HeLa cells could contain epigenetically silenced genomic loci less prone to tether the CRISPR/Cas9 complex compared to viral derived cDNA, the high frequency of indels occurring in the transgene strongly supports the specificity of the designed gRNAs.

The major drawback of the CRISPR/Cas9 system is the possibility to induce genome-wide unwanted off-target effects. The COSMID webtool[35] predicted 28 (Table S1) and six putative genome-wide off-targets for gRNA1 and gRNA5, respectively. We investigated, by NGS, the 10 top-ranked off-targets predicted for gRNA1 mapping to intragenic regions and all the potential off-targets predicted for gRNA5 (Figure 1D). Briefly, a Hygromycin (Hygro) resistance cassette under the control of TK promoter was cloned in both effector plasmids (SpCas9_gRNA1-TKHygro and VQRHF1-SpCas9_gRNA5-TKHygro) to analyze the off-target sites in Hygro-selected non-clonal HEK293T cells expressing Cas9 nuclease and gRNA. Targeted deep sequencing detected cleavage above background in the intronic sequences of four genes: three predicted for gRNA1 and one for gRNA5 (Figure 1D). The CRISPResso analysis of potential splice site modifications predicted no risk of interference with the canonical splicing signals of the hit introns (Figure S3A). Indeed, the expression of the cleaved genes in human immortalized retinal pigment epithelium (hRPE) cells[41] transfected with SpCas9_gRNA1 or VQRHF1-SpCas9_gRNA5 was not perturbed by the intronic editing (Figures S3B and S3C).

p.Pro347Ser-specific editing leads to efficient degradation of RHO variants

To better characterize the RHO variants generated upon gene editing, we analyzed the frequency and the type of indels scored by CRISPResso in Pro347Ser RHO HeLa clones transfected with SpCas9_gRNA1 or VQRHF1-SpCas9_gRNA5 and we investigated whether small insertions or deletions created by NHEJ repair involving the last codons of c.1039C>T *RHO* cDNA would generate frameshift changes that in turn would knock out this allele. The analysis, set for a −10 +10 window surrounding the target sites, revealed that the most frequent indels generated by gRNA1 were 1–2 nt deletions occurring around positions −2 to +1 relative to the cleavage site (Figure 1E). Conversely, the insertion of a T nucleotide in the cut site was the top-ranked indel in gRNA5-treated samples (Figure 1F). To refine the analysis further, we calculated the frequency of deletions, substitutions, and insertions in the gRNA1- and gRNA5-treated samples. The results showed that deletions were the most frequent type of modifications for both gRNAs (88.9% and 47.8% of all indels, Figure 1G) but, interestingly, a higher prevalence of substitutions and insertions were scored in the gRNA5 (32.8% and 19.4% of all indels)-treated samples compared to gRNA1 (9.9% and 1.9% of all indels)-treated samples (Figure 1G). The analysis of indel distribution revealed extended deletions, up to 80 nt, and insertions, up to 13 nt, in samples treated with SpCas9_gRNA1 or VQRHF1-SpCas9_gRNA5 (Figure S4). More importantly, despite the differences in the type of indels and their distribution, 81.5% and 68.9% of all edited sequences lead to frameshift alterations (Figure 1H), suggesting a potentially favorable outcome of p.Pro347Ser RHO knockdown.

Since Cas9-mediated editing is occurring in the RHO C terminus, a comprehensive *in vitro* study of the localization and degradation of RHO variants generated upon editing was performed. The six most frequent indels identified by CRISPResso upon gRNA1- or gRNA5-mediated editing resulted in shifted reading frame (delG, delGG, and insT, Figure S5) or in-frame deletion of a region including the TAA stop codon and generation of new termination codons downstream of the canonical one (del9, del12.1, del12.5, Figure S5).

Cellular localization of these six RHO variants was investigated by immunofluorescence in CHO cells transfected with plasmids expressing the RHO variants fused at the N-terminal region to a human influenza hemagglutinin (HA) tag. All these variants showed localization of RHO at the plasma membrane (Figure 2A), as observed for the WT and p.Pro347Ser rhodopsin (Figure S6), but distinct from p.Pro23His RHO, which is retained in the endoplasmic reticulum (ER).[42] The plasma membrane localization was also quantified by "In-Cell Western" assay on non-permeabilized and permeabilized CHO cells transfected with the selected six RHO variants. The assay confirmed the almost complete localization of all analyzed variants to the plasma membrane, as well as the ER retention of p.Pro23His rhodopsin (Figures S7A and S7B). Persistent expression of rhodopsin variants generated upon editing could impair the therapeutic benefits of this strategy. Therefore, the expression of WT, p.Pro347Ser, and the selected six RHO variants was evaluated by immunoblot

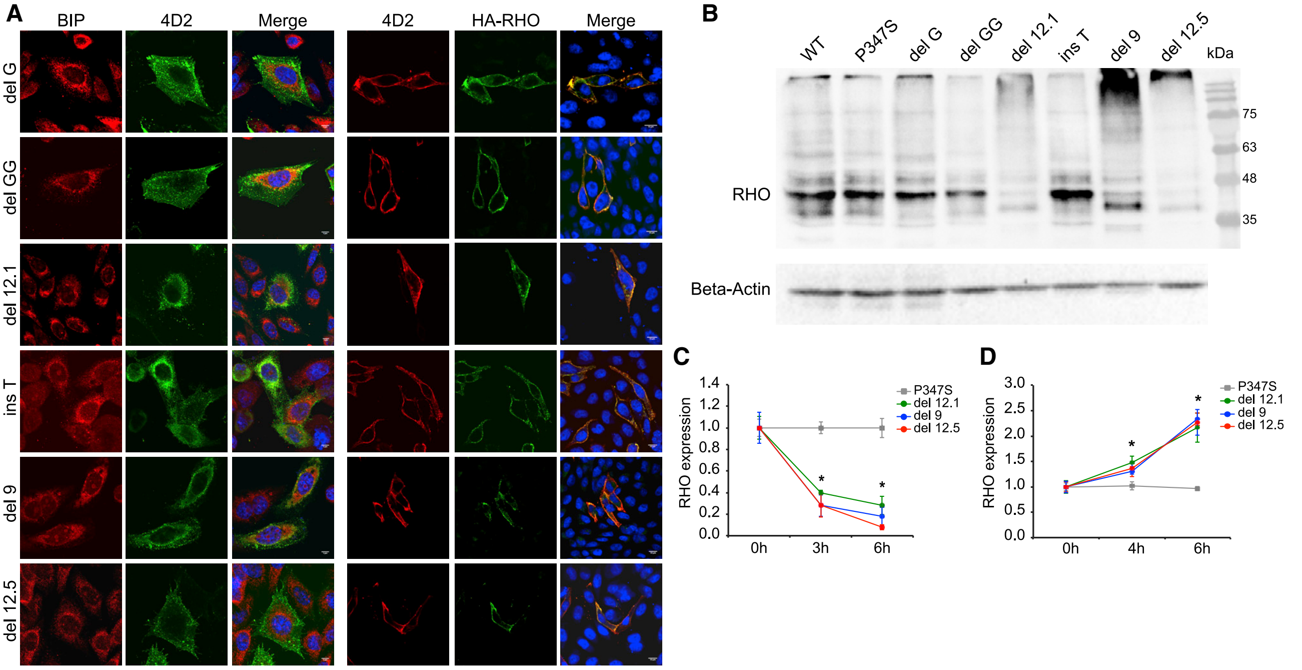

Figure 2. Biochemical characterization of the most frequent RHO variants generated after editing
(A) Immunofluorescence analysis of CHO cells transfected with plasmids coding for RHO variants. Permeabilized cells (left) were stained with anti-BIP and anti-4D2 antibodies, and the scale bar represents 5 μm. Cells not permeabilized (right) were stained with anti-4D2 and anti-HA antibodies, and the scale bar represents 10 μm.
(B) Immunoblot analysis of WT, p.Pro347Ser, and most frequent RHO variants generated after editing expressed in CHO cells transfected with the respective coding plasmids. Anti-HA antibody was used to detect rhodopsin (RHO). The immunoblotting was normalized with an anti-beta-actin antibody.
(C) Densitometric analysis of immunoblots performed on CHO cells transfected with plasmids coding for p.Pro347Ser, del9, del12.1, and del12.5 RHO variants and treated with 10 μg/mL cycloheximide (CHX). The experiment was performed in triplicate and is presented as mean ± SEM. *p value < 0.05.
(D) Densitometric analysis of immunoblots performed on CHO cells transfected with plasmids coding for p.Pro347Ser, del9, del12.1, and del12.5 RHO variants and treated with 50 μM MG-132 proteasome inhibitor. The experiment was performed in triplicate and is presented as mean ± SEM. *p value < 0.05.

in CHO cells 2 days after transfection (Figure 2B and Figure S7C). The rate of RHO variant degradation in transfected CHO cells was measured upon treatment with the translation inhibitor cycloheximide (CHX). Compared to p.Pro347Ser rhodopsin, rhodopsin proteins carrying 9- and 12-nt deletions were rapidly degraded, with between 60%–95% removed after 6 h of translation inhibition (Figure 2C). Treatment with proteasome inhibitor MG-132 at different time points (0, 4, 6 h) revealed that the degradation of del9, del12.1, and del12.5 rhodopsin variants was proteasome mediated (Figure 2D). The degradation of the most frequent variants generated by Cas9 editing demonstrates the desired robust reduction of rhodopsin observed in Pro347Ser RHO HeLa cells transfected with SpCas9_gRNA1 or VQRHF1-SpCas9_gRNA5 effector plasmids. Similarly, p.Pro347Ser RHO protein was reduced to 40% upon transfection of effector plasmids in Pro347Ser RHO HeLa cells (Figures 3A and 3B). Furthermore, the transcript coding for p.Pro347Ser RHO was also significantly downregulated after editing as demonstrated by quantitative reverse-transcriptase PCR (qRT-PCR) (Figure 3C). Control plasmids did not influence the expression of p.Pro347Ser RHO and, notably, WT rhodopsin was not perturbed by the treatment with SpCas9_gRNA1 or VQRHF1-SpCas9_gRNA5, as expected by gene editing occurring specifically on the c.1039C>T *RHO* allele (Figures 3A–3C).

Interestingly, expression of Pro347Ser RHO in the corresponding HeLa clone induced higher cytotoxicity than WT RHO (Figure 3D). Indeed, an LDH cytotoxicity assay showed that the Pro347Ser RHO HeLa clone had lower viability (90%) than the WT RHO HeLa clone (99%). Following treatment with Cas9 and specific gRNA, there was a 50%–60% increase in cell viability compared to negative controls, suggesting that approximately 60% of knockdown of p.Pro347Ser RHO protein was sufficient to significantly reduce the toxic effects of this variant *in vitro* (Figure 3D).

Allele-specific editing significantly reduces p.Pro347Ser RHO expression in mouse photoreceptors

To translate these *in vitro* findings to a preclinical model of RP, we treated transgenic mice carrying the human *RHO* allele with the p.Pro347Ser variant.[14] This model carries the two wild-type murine *Rho* alleles as well as an undefined number of transgenic alleles with the p.Pro347Ser variant and has a 1:1 ratio of transgene to endogenous opsin

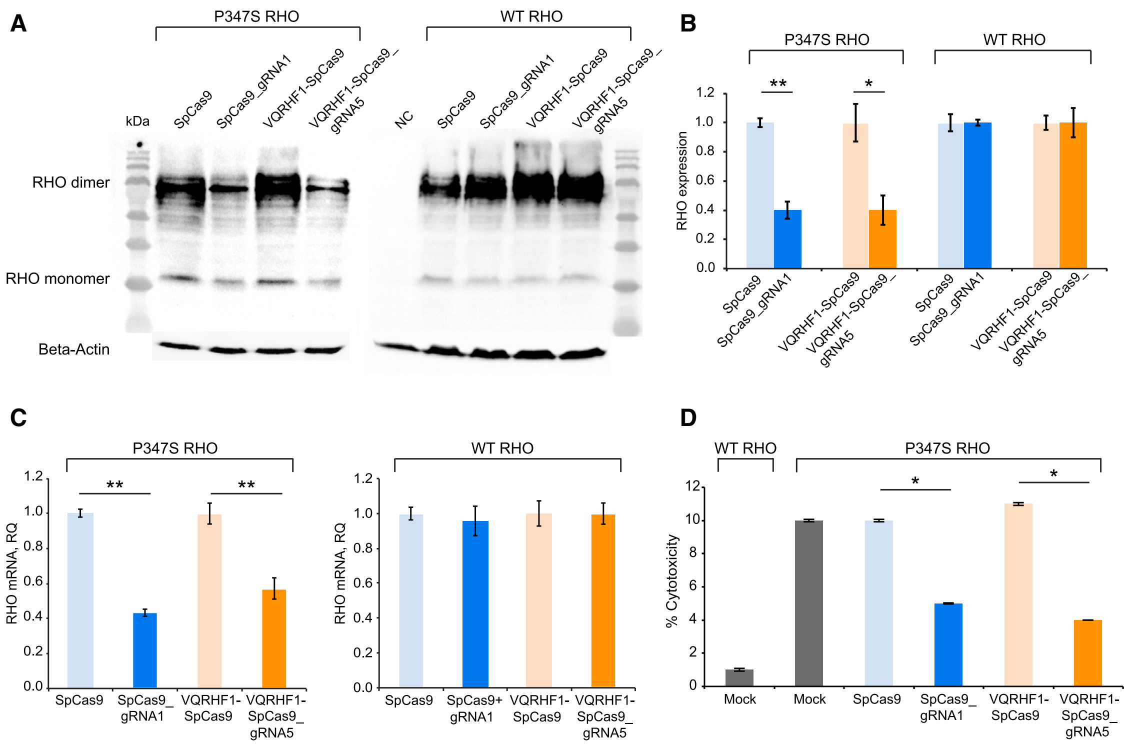

Figure 3. Efficient knockdown of p.Pro347Ser RHO expression *in vitro*

(A) Immunoblot for rhodopsin protein expressed in Pro347Ser and WT RHO HeLa clones transfected with SpCas9_gRNA1 and VQRHF1-SpCas9_gRNA5 plasmids and control plasmids (SpCas9 and VQRHF1-SpCas9). 4D2 antibody was used to detect rhodopsin. The immunoblotting was normalized with anti-beta-actin antibody.

(B) Densitometric quantification of rhodopsin protein level normalized to beta-actin after editing. The experiment was performed in triplicate and is presented as mean ± SEM. *$p < 0.05$; **$p < 0.01$.

(C) Pro347Ser and WT RHO HeLa clones were transfected with SpCas9_gRNA1 and VQRHF1-SpCas9_gRNA5 plasmids and control plasmids (SpCas9 and VQRHF1-SpCas9). The relative quantity (RQ) was calculated with the $2^{-\Delta\Delta CT}$ quantification and is reported in the y axis. Each sample was run in triplicate. **$p < 0.01$.

(D) Lactate dehydrogenase (LDH) assay of Pro347Ser RHO HeLa clone transfected with SpCas9_gRNA1 and VQRHF1-SpCas9_gRNA5 plasmids and control plasmids (SpCas9 and VQRHF1-SpCas9). Mock-transfected Pro347Ser RHO HeLa cells and WT RHO HeLa cells were used as positive and negative controls. The experiment was performed in triplicate and is presented as mean ± SEM. *p value < 0.05.

mRNA, which causes severe retinal degeneration by postnatal day (P) 30, as seen by electroretinogram responses and loss of outer nuclear layer (ONL) thickness.[14] To improve the translational potential of our approach, CRISPR/Cas9 components were packaged into AAV2/8 vectors. AAV delivery of the CRISPR/Cas9 system or therapeutic genes has been proven very successful for the treatment of retinal diseases in various preclinical studies.[43–45] To restrict the expression of SpCas9 to photoreceptors, we employed the interphotoreceptor retinoid-binding protein (IRBP) promoter, while GFP expression, driven by the *RHO* promoter, tracked the expression of gRNAs in injected eyes. Briefly, effector vector combinations were AAV expressing WT or VQRHF1 SpCas9 with AAV expressing gRNA1 or gRNA5, while control AAV vector combinations were SpCas9 with AAV expressing gRNA scramble (Figure 4A). Pro347Ser transgenic mice received a single subretinal injection per eye at P7 of two AAV2/8 vectors carrying either WT or VQRHF1 SpCas9 in combination with either effector or scramble gRNA. Four weeks after injection (P40), molecular analyses of injected retinae showed co-expression of SpCas9, WT or VQRHF1, GFP, and *Phosphodiesterase 6b* (*Pde6b*) genes (Figure S8), indicating that the AAV vectors targeted photoreceptors upon subretinal injection. Then, the frequency and type of indels in the target locus and the inactivation of the transcript coding for p.Pro347Ser RHO were evaluated. NGS analysis detected *RHO* indels up to 14% in 11 retinae derived from SpCas9+gRNA1 treatment and up to 30% in 15 retinae from VQRHF1-SpCas9+gRNA5 treatment (Figure 4B). No *RHO* indels were detected in the retinae treated with the control vectors ($n = 6$, data not shown). The most common alterations on target sites were insertions and deletions that lead to frameshift as observed *in vitro* (Figure 4C), suggesting destabilization of p.Pro347Ser *RHO* transcripts and protein, which has the potential to provide therapeutic benefit to photoreceptor degeneration. Our strategy specifically targeting the human p.Pro347Ser-coding *RHO* gene resulted in a significant reduction, from 20% to 60%, of the c.1039C>T mRNA in 11 out of 20 retinae expressing the effector vectors compared to the contralateral retinae injected with control vectors (Figure 4D).

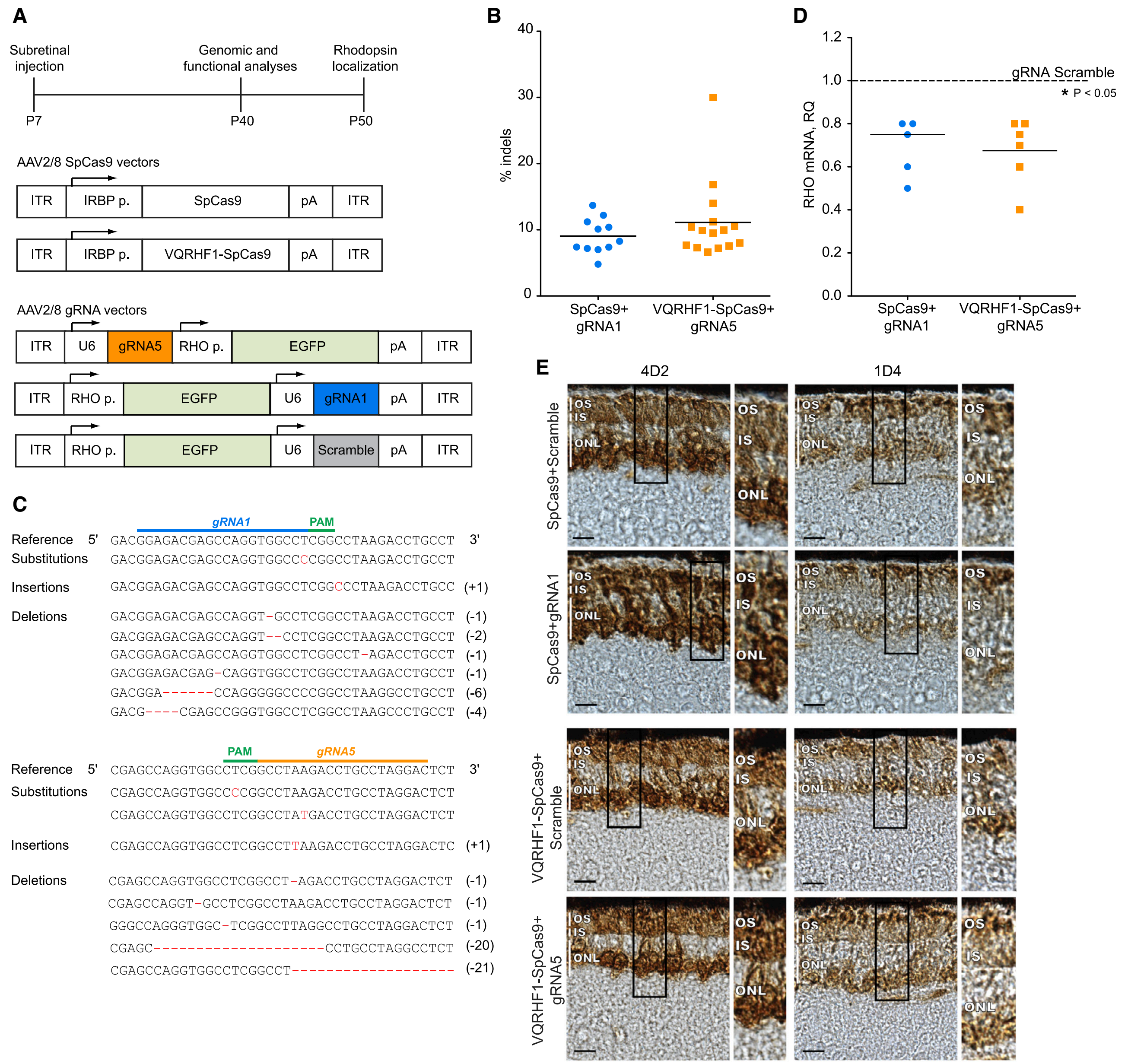

Figure 4. Allele-specific editing in mouse photoreceptors

(A) (Top) a scheme of the experimental timeline is depicted; (middle) AAV2/8 vectors expressing the WT or VQRHF1 SpCas9 under the control of IRBP promoter are schematized; (bottom) AAV2/8 vectors expressing the gRNA (gRNA1, gRNA5, or scramble) and the GFP under the control of *RHO* promoter.

(B) Indels frequency determined by NGS in retinae injected with effector vectors.

(C) Representation of indels scored in retinae treated with gRNA1 or gRNA5 AAV vector coupled to appropriate AAV-SpCas9 vector. Nucleotides inserted or deleted are reported on the right.

(D) Downregulation of *RHO* transcript coding for p.Pro347Ser RHO in retinae injected with effector vectors with respect to retinae treated with scramble vectors. The averages are depicted with a bar. *p < 0.05.

(E) Rhodopsin localization was investigated at P50 in retinae injected with effector vectors using 4D2 and 1D4 antibodies against rhodopsin. Representative images are shown. Scale bar represents 10 μm. Zoomed areas of photoreceptors are shown on the side. OS, outer segment; IS, inner segment; ONL, outer nuclear layer.

The localization of RHO was investigated at P50 in retinae injected with the effector and control vectors (Figure 4E). In retinae injected with control vectors, total RHO (murine WT RHO + human p.Pro347Ser RHO) was detected using the 4D2 antibody targeting the N terminus of the protein, which would recognize both the WT and C-terminal RHO variant. The predominant localization was observed in the ONL and, to a lesser extent, in the inner segment (IS) and outer segment (OS). By contrast, in retinae injected with effector vectors that downregulate the expression of the dominant human p.Pro347Ser RHO, RHO was more evident in the IS and OS, suggesting

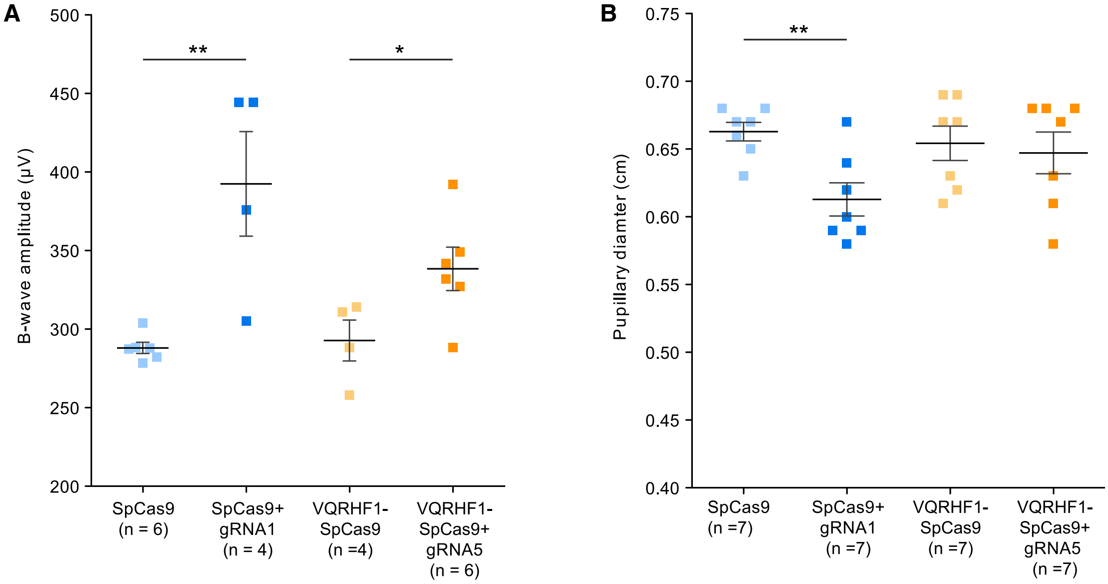

Figure 5. Significant improvement of retinal electrical function and pupillary light response
(A and B) Pro347Ser transgenic mice injected with effector or control AAV2/8 vectors were examined at P40 by ERG (A), as shown in the data point distribution of B-wave amplitude at 20 cd.s/m^2, and PLR analysis (B). Individual eyes are depicted as squares. Data are presented as mean ± SEM. *$p < 0.05$; **$p < 0.01$.

the restoration of a correct localization to the OS. To extend these data, endogenous murine WT rhodopsin was stained using the 1D4 antibody that recognizes an epitope in rhodopsin C-terminal region, which includes the Pro347 residue, and does not react with the Ser347 protein. In retinae transduced with control vectors, the predominant localization of murine RHO was identified in the rod cell body in the ONL, along the IS, and also in the OS. Transduction with effector vectors rescued the localization of endogenous WT rhodopsin primarily to the OS. Staining for GFP in both control and treated eyes identified the transduced portion of the retina (Figure S9).

CRISPR/Cas9-mediated specific *RHO* allele knockdown in Pro347Ser mice

We then investigated whether c.1039C>T *RHO* allele-specific knockdown has a therapeutic efficacy benefit on visual function. The retinal function of Pro347Ser transgenic mice treated with AAV effector and control vectors was examined 1 month after injection (P40) by both electroretinography (ERG) and PLRs. ERG analysis showed significant improvement of the b-wave amplitudes at 20 cd.s/m^2 in retinae treated with either SpCas9+gRNA1 or VQRHF1-SpCas9+gRNA5 compared to their corresponding controls (Figure 5A). The improvement in retinal electrical activity was mirrored by post-photoreceptor responses to light stimuli, which result in transient pupil constriction. The eyes injected with SpCas9+gRNA1 AAVs showed significantly greater pupillary constriction than gRNA scramble-injected eyes (Figure 5B). In contrast, the eyes injected with VQRHF1-SpCas9+gRNA5 AAVs showed more variation in their pupillary constriction that was not statistically significantly different compared to their control group.

Taken together, these results indicate that a precise editing approach to selectively silence a dominant disease-associated variant in *RHO* holds promise for slowing down the photoreceptor degeneration observed in dominant RPs.

Discussion

RP causes the progressive death of photoreceptors and eventually blindness. The treatment of RP is challenging, but an early therapeutic intervention aimed at blocking or reducing rod degeneration would be an effective approach to preserve vision in individuals affected by RP. This was the aim of earlier nutrient trials in RP, and results were assessed over several years.[46–48]

More recently, gene therapy approaches aimed at correcting disease-associated variants identified in genes causing RPs have been reported.[4,49,50] In this study, we addressed a CRISPR/Cas9-mediated gene editing approach for a common variant in *RHO* that causes autosomal dominant RP (adRP) (RP4 [MIM: 613731]). The ClinVar Miner database[51] lists 156 variants in *RHO*, and at least 50 of them are pathogenic or likely pathogenic variants associated with RP4. In this scenario, the knockdown of both alleles followed by gene supplementation represents the most cost-effective approach to pursue. Indeed, the ablate-and-replace strategy could be used for the treatment of all *RHO* disease-associated variants, thus circumventing the allelic heterogeneity of the disease. However, in the case of editing, this strategy requires bi-allelic events, a

difficult goal to achieve *in vivo*. Moreover, the threshold level of RHO protein in the cell represents a crucial issue. For rhodopsin, there is a fine balance between insufficiency and toxicity[52,53] that requires a fine-tuning of the replacement or augmentation of WT rhodopsin. Indeed, the "suppression and replacement" approach involves the risk of converting a dominant condition to a recessive RP should replacement not be as effective as suppression. Moreover, an excess of RHO expressed by an exogenous transgene cassette could exert a detrimental effect for photoreceptor cells, as shown by Mao et al., who described that WT RHO overexpression leads to retinal degeneration in WT mice.[54]

Conversely, a specific and permanent silencing of the variant allele by CRISPR/Cas9, as proposed in this study, would prevent the pathogenic effects of most dominant variants while preserving the WT allele, which would also not require the ethical issue of disrupting a functional human gene. Indeed, allele-specific knockout would address both dominant-negative and gain-of-function changes, while conventional gene replacement therapy is indicated for haploinsufficiency. Notably, haploinsufficiency has been linked to adRP caused by disease-associated variants in *PRPF3* or *PRPH2* but not in *RHO*.[6,23]

Several studies demonstrated beneficial effects of CRISPR/Cas9 on adRP treatment in preclinical models.[12,21,55] The majority of the reported studies were designed to correct Pro23His allele, the most frequent disease-associated variant accounting for ~10% of the adRP cases in North America. A gene-editing strategy focused on C-terminal domain of human RHO has not been described and, in particular, the correction of the p.Pro347Ser variant was never addressed. Here, we report a CRISPR/Cas9-mediated knockdown of the c.1039C>T transition, a dominant variant leading to severe adRP that is prevalent in the European population. SpCas9 and its high-fidelity variant (VQRHF1), combined with allele-specific gRNAs, were employed to knock down the p.Pro347Ser variant in engineered HeLa cell lines and in Pro347Ser transgenic mice. The *in vitro* experiments demonstrated that both SpCas9 variants, WT and VQRHF1 SpCas9, reached efficient and allele-specific editing, although the high-fidelity variant resulted in a safer profile with just one genome-wide cleaved predicted off-target site without detectable effect on the expression of the off-target gene. Notably, the genomic analysis of indels was instrumental to predict the fate of the most frequent events upon CRISPR/Cas9 editing. Double strand breaks tailored to a dominant variant and repaired by NHEJ could, in principle, lead to new rhodopsin variants that exert a toxic effect on their own or a dominant-negative effect on the WT protein. A detailed *in vitro* characterization of the most frequent changes generated after editing demonstrated a proteasome-mediated degradation of the most-common frameshifted indels and intriguingly, the in-frame variants carrying a longer cytoplasmic tail. Indeed, RHO variants localized to the plasma membrane could be degraded by endocytic protein quality control mechanism occurring for non-native plasma membrane protein.[56] These results support the potential safety of the gene editing in the C-terminal domain of rhodopsin, broadening the application of CRISPR-mediated editing to the 3′ terminus of genes. Besides the safety issue, allele-specific editing performed *in vitro* demonstrated a robust knockdown of p.Pro347Ser RHO expression that significantly improved the viability of cells stably expressing the p.Pro347Ser rhodopsin, supporting the idea that degradation of p.Pro347Ser RHO protein *in vivo* could ameliorate the RP phenotype. This is reminiscent of the cytotoxicity observed in photoreceptors expressing either this or other RHO variants causing adRP. Class I variants, which include Pro347Ser, cause improper trafficking of RHO to the OS of the photoreceptor; however, the mechanisms of cell death are still unknown. Various mechanisms for the induction of apoptosis by class I variants have been proposed, including the impairment of vesicular trafficking to the OS and plasma membrane, the metabolic burden caused by the continuous degradation of the mis-trafficked RHO, induction of the unfolded protein response, and the interference of RHO variants' being present in the cell membrane with cellular processes including intracellular signaling. The mechanisms of increased cell death in the Pro347Ser RHO HeLa model are not clear, and some of the processes reported *in vivo* may not be relevant in this cell culture model. Nevertheless, the cell model could be useful to probe mechanisms in the future and appeared to be specific to Pro347Ser expression because the Pro347Ser RHO HeLa clone showed improved cell viability following gene editing and RHO knockdown.

To test this hypothesis, we transferred the CRISPR/Cas9 editing platform to the retina of Pro347Ser transgenic mice using AAV2/8. Although Pro347Ser transgenic mice do not match the copy number or genomic context of the p.Pro347Ser RHO variant in individuals affected by RP, they represent a valuable *in vivo* model to test the efficacy of gene therapy. AAV2/8 vectors efficiently transfer genes to photoreceptors in the retina,[31,49,57] and successful applications of CRISPR-AAV vectors in retinal diseases are already reported.[58–60] Pro347Ser transgenic mice received a single injection of either effector or control AAV2/8 vector combinations. One month later, during rod degeneration, molecular analyses revealed a variable but effective permanent knockdown of human c.1039C>T transcript as a consequence of allele-specific gene editing provided by both SpCas9 variants. The frequency of indels significantly differs from that scored *in vitro*. This could be due to a lower transduction efficiency with respect to the transfection of HeLa cells, additional copies of the Pro347Ser allele in the transgenic mice or to the requirement of two AAVs to reconstitute the effector SpCas9/gRNA system. The most frequent indels types were insertions and deletions that lead to frameshift as observed *in vitro*, suggesting a comparable outcome for p.Pro347Ser rhodopsin protein *in vivo*. Significant evidence of therapeutic benefit was

obtained using both PLRs and ERG. ERG comparisons between treated and control eyes at P40 demonstrated significantly improved responses in treated eyes with respect to the controls.

The beneficial effects observed using ERG were also observed at P50 using histological analyses on treated and control retinae. The mislocalization and retention of rhodopsin in the ONL was partially rescued in retinae injected with effector AAV2/8 vectors, showing an increased localization of WT murine rhodopsin to OS as consequence of downregulation of the dominant effect exerted by human p.Pro347Ser RHO. It is noteworthy that although the ONL structure was not significantly improved by the treatment, a significant functional recovery by ERG and PLR was registered, indicating that the reduction of human *RHO* transcript coding for p.Pro347Ser was sufficient to improve the function of surviving rods but did not significantly prevent their death.

Overall, our study provides proof of concept for CRISPR/Cas9-mediated allele-specific targeting of a common *RHO* variant associated with adRP. Whether using the WT or the VQRHF1 SpCas9 variant, which show here similar efficiency, it could be beneficial for 62% of the pathogenic and likely pathogenic variants in *RHO*, listed in ClinVar Miner, displaying genomic sequences suitable for allele-specific inactivation (Table S2), with no need for RHO supplementation therapy. The translation of this genome editing approach to the clinic will require further pre-clinical testing including using affected-individual-derived cell lines carrying one WT and one Pro347Ser variant RHO allele, potentially through human retinal organoids to model the variant editing in right genomic and cellular context. Moreover, this CRISPR/Cas9 approach would benefit from the development of more efficient clinically relevant non-toxic viral or non-viral delivery systems allowing the targeting and editing of a higher number of photoreceptors. Allele-specific genome editing therefore has the potential to become the therapeutic intervention of choice for precise silencing of genetic variants causing dominant retinal degeneration.

Data and code availability

The NGS datasets supporting the current study have not been deposited in a public repository but are available from the corresponding author (A.R.) on request.

Supplemental information

Supplemental information can be found online at https://doi.org/10.1016/j.ajhg.2021.01.006.

Acknowledgments

The authors are grateful to Valeria Marigo (University of Modena and Reggio Emilia) for helpful discussion throughout the development of this study. The authors wish to thank the LaBSSAH - CIBIO Next Generation Sequencing Facility of the University of Trento for sequencing samples and Robert Molday (Department of Biochemistry and Molecular Biology, University of British Columbia, Canada) for the gift of the anti-Rho-1D4 antibody and Idil Ahmed (Department of Eye Pathology, UCL Institute of Ophthalmology) for the support in performing the histology. This work was supported by Fondazione Roma, Italy (call for Retinitis Pigmentosa) to A.R.; by the ERC Advanced grant “EYEGET” (694323), the European Union Horizon 2020 “Upgrade” (825825), and the Foundation Fighting Blindness grant TA-GT-0619-0762 to A.A.; and by grants from the Wellcome Trust, United Kingdom (205041) and the Foundation Fighting Blindness USA (PPA-0717-0719-RAD) to M.E.C.

Declaration of interests

C.P., M.L., D.B., A.A., and A.R. are listed as inventors on a patent application related to this work.

Received: August 31, 2020
Accepted: January 8, 2021
Published: January 27, 2021

Web resources

Addgene, https://www.addgene.org
ClinVar Miner, https://clinvarminer.genetics.utah.edu
CRISPResso web tool, https://crispresso.pinellolab.partners.org/
Genetic home reference NIH, https://medlineplus.gov/genetics/
OMIM, https://omim.org/
RetNet - Retinal Information Network, https://sph.uth.edu/retnet

References

1. Verbakel, S.K., van Huet, R.A.C., Boon, C.J.F., den Hollander, A.I., Collin, R.W.J., Klaver, C.C.W., Hoyng, C.B., Roepman, R., and Klevering, B.J. (2018). Non-syndromic retinitis pigmentosa. Prog. Retin. Eye Res. *66*, 157–186.
2. Hamel, C. (2006). Retinitis pigmentosa. Orphanet J. Rare Dis. *1*, 40.
3. Parmeggiani, F. (2011). Clinics, epidemiology and genetics of retinitis pigmentosa. Curr. Genomics *12*, 236–237.
4. Benati, D., Patrizi, C., and Recchia, A. (2020). Gene editing prospects for treating inherited retinal diseases. J. Med. Genet. *57*, 437–444.
5. Dias, M.F., Joo, K., Kemp, J.A., Fialho, S.L., da Silva Cunha, A., Jr., Woo, S.J., and Kwon, Y.J. (2018). Molecular genetics and emerging therapies for retinitis pigmentosa: Basic research and clinical perspectives. Prog. Retin. Eye Res. *63*, 107–131.
6. Athanasiou, D., Aguila, M., Bellingham, J., Li, W., McCulley, C., Reeves, P.J., and Cheetham, M.E. (2018). The molecular and cellular basis of rhodopsin retinitis pigmentosa reveals potential strategies for therapy. Prog. Retin. Eye Res. *62*, 1–23.
7. Cideciyan, A.V., Jacobson, S.G., Drack, A.V., Ho, A.C., Charng, J., Garafalo, A.V., Roman, A.J., Sumaroka, A., Han, I.C., Hochstedler, M.D., et al. (2019). Effect of an intravitreal antisense oligonucleotide on vision in Leber congenital amaurosis due to a photoreceptor cilium defect. Nat. Med. *25*, 225–228.
8. Concepcion, F., Mendez, A., and Chen, J. (2002). The carboxyl-terminal domain is essential for rhodopsin transport in rod photoreceptors. Vision Res. *42*, 417–426.

9. Deretic, D., Schmerl, S., Hargrave, P.A., Arendt, A., and McDowell, J.H. (1998). Regulation of sorting and post-Golgi trafficking of rhodopsin by its C-terminal sequence QVS(A)PA. Proc. Natl. Acad. Sci. USA *95*, 10620–10625.
10. Berson, E.L., Rosner, B., Weigel-DiFranco, C., Dryja, T.P., and Sandberg, M.A. (2002). Disease progression in patients with dominant retinitis pigmentosa and rhodopsin mutations. Invest. Ophthalmol. Vis. Sci. *43*, 3027–3036.
11. LaVail, M.M., Nishikawa, S., Steinberg, R.H., Naash, M.I., Duncan, J.L., Trautmann, N., Matthes, M.T., Yasumura, D., Lau-Villacorta, C., Chen, J., et al. (2018). Phenotypic characterization of P23H and S334ter rhodopsin transgenic rat models of inherited retinal degeneration. Exp. Eye Res. *167*, 56–90.
12. Li, P., Kleinstiver, B.P., Leon, M.Y., Prew, M.S., Navarro-Gomez, D., Greenwald, S.H., Pierce, E.A., Joung, J.K., and Liu, Q. (2018). Allele-Specific CRISPR-Cas9 Genome Editing of the Single-Base P23H Mutation for Rhodopsin-Associated Dominant Retinitis Pigmentosa. CRISPR J *1*, 55–64.
13. Berson, E.L., Rosner, B., Sandberg, M.A., and Dryja, T.P. (1991). Ocular findings in patients with autosomal dominant retinitis pigmentosa and a rhodopsin gene defect (Pro-23-His). Arch. Ophthalmol. *109*, 92–101.
14. Li, T., Snyder, W.K., Olsson, J.E., and Dryja, T.P. (1996). Transgenic mice carrying the dominant rhodopsin mutation P347S: evidence for defective vectorial transport of rhodopsin to the outer segments. Proc. Natl. Acad. Sci. USA *93*, 14176–14181.
15. Greenwald, D.L., Cashman, S.M., and Kumar-Singh, R. (2013). Mutation-independent rescue of a novel mouse model of Retinitis Pigmentosa. Gene Ther. *20*, 425–434.
16. Millington-Ward, S., Chadderton, N., O'Reilly, M., Palfi, A., Goldmann, T., Kilty, C., Humphries, M., Wolfrum, U., Bennett, J., Humphries, P., et al. (2011). Suppression and replacement gene therapy for autosomal dominant disease in a murine model of dominant retinitis pigmentosa. Mol. Ther. *19*, 642–649.
17. Gorbatyuk, M., Justilien, V., Liu, J., Hauswirth, W.W., and Lewin, A.S. (2007). Preservation of photoreceptor morphology and function in P23H rats using an allele independent ribozyme. Exp. Eye Res. *84*, 44–52.
18. Cideciyan, A.V., Sudharsan, R., Dufour, V.L., Massengill, M.T., Iwabe, S., Swider, M., Lisi, B., Sumaroka, A., Marinho, L.F., Appelbaum, T., et al. (2018). Mutation-independent rhodopsin gene therapy by knockdown and replacement with a single AAV vector. Proc. Natl. Acad. Sci. USA *115*, E8547–E8556.
19. Mussolino, C., Sanges, D., Marrocco, E., Bonetti, C., Di Vicino, U., Marigo, V., Auricchio, A., Meroni, G., and Surace, E.M. (2011). Zinc-finger-based transcriptional repression of rhodopsin in a model of dominant retinitis pigmentosa. EMBO Mol. Med. *3*, 118–128.
20. Botta, S., Marrocco, E., de Prisco, N., Curion, F., Renda, M., Sofia, M., Lupo, M., Carissimo, A., Bacci, M.L., Gesualdo, C., et al. (2016). Rhodopsin targeted transcriptional silencing by DNA-binding. eLife *5*, e12242.
21. Giannelli, S.G., Luoni, M., Castoldi, V., Massimino, L., Cabassi, T., Angeloni, D., Demontis, G.C., Leocani, L., Andreazzoli, M., and Broccoli, V. (2018). Cas9/sgRNA selective targeting of the P23H Rhodopsin mutant allele for treating retinitis pigmentosa by intravitreal AAV9.PHP.B-based delivery. Hum. Mol. Genet. *27*, 761–779.
22. Marraffini, L.A. (2015). CRISPR-Cas immunity in prokaryotes. Nature *526*, 55–61.
23. Diakatou, M., Manes, G., Bocquet, B., Meunier, I., and Kalatzis, V. (2019). Genome Editing as a Treatment for the Most Prevalent Causative Genes of Autosomal Dominant Retinitis Pigmentosa. Int. J. Mol. Sci. *20*, 2542.
24. Dryja, T.P., McGee, T.L., Reichel, E., Hahn, L.B., Cowley, G.S., Yandell, D.W., Sandberg, M.A., and Berson, E.L. (1990). A point mutation of the rhodopsin gene in one form of retinitis pigmentosa. Nature *343*, 364–366.
25. Fernandez-San Jose, P., Blanco-Kelly, F., Corton, M., Trujillo-Tiebas, M.J., Gimenez, A., Avila-Fernandez, A., Garcia-Sandoval, B., Lopez-Molina, M.I., Hernan, I., Carballo, M., et al. (2015). Prevalence of Rhodopsin mutations in autosomal dominant Retinitis Pigmentosa in Spain: clinical and analytical review in 200 families. Acta Ophthalmol. *93*, e38–e44.
26. Kleinstiver, B.P., Prew, M.S., Tsai, S.Q., Topkar, V.V., Nguyen, N.T., Zheng, Z., Gonzales, A.P., Li, Z., Peterson, R.T., Yeh, J.R., et al. (2015). Engineered CRISPR-Cas9 nucleases with altered PAM specificities. Nature *523*, 481–485.
27. Kleinstiver, B.P., Pattanayak, V., Prew, M.S., Tsai, S.Q., Nguyen, N.T., Zheng, Z., and Joung, J.K. (2016). High-fidelity CRISPR-Cas9 nucleases with no detectable genome-wide off-target effects. Nature *529*, 490–495.
28. Olsson, J.E., Gordon, J.W., Pawlyk, B.S., Roof, D., Hayes, A., Molday, R.S., Mukai, S., Cowley, G.S., Berson, E.L., and Dryja, T.P. (1992). Transgenic mice with a rhodopsin mutation (Pro23His): a mouse model of autosomal dominant retinitis pigmentosa. Neuron *9*, 815–830.
29. Latella, M.C., Di Salvo, M.T., Cocchiarella, F., Benati, D., Grisendi, G., Comitato, A., Marigo, V., and Recchia, A. (2016). In vivo Editing of the Human Mutant Rhodopsin Gene by Electroporation of Plasmid-based CRISPR/Cas9 in the Mouse Retina. Mol. Ther. Nucleic Acids *5*, e389.
30. Ohgane, K., Dodo, K., and Hashimoto, Y. (2010). Retinobenzaldehydes as proper-trafficking inducers of folding-defective P23H rhodopsin mutant responsible for retinitis pigmentosa. Bioorg. Med. Chem. *18*, 7022–7028.
31. Allocca, M., Mussolino, C., Garcia-Hoyos, M., Sanges, D., Iodice, C., Petrillo, M., Vandenberghe, L.H., Wilson, J.M., Marigo, V., Surace, E.M., and Auricchio, A. (2007). Novel adeno-associated virus serotypes efficiently transduce murine photoreceptors. J. Virol. *81*, 11372–11380.
32. Doria, M., Ferrara, A., and Auricchio, A. (2013). AAV2/8 vectors purified from culture medium with a simple and rapid protocol transduce murine liver, muscle, and retina efficiently. Hum. Gene Ther. Methods *24*, 392–398.
33. Drittanti, L., Rivet, C., Manceau, P., Danos, O., and Vega, M. (2000). High throughput production, screening and analysis of adeno-associated viral vectors. Gene Ther. *7*, 924–929.
34. Chapple, J.P., Hardcastle, A.J., Grayson, C., Spackman, L.A., Willison, K.R., and Cheetham, M.E. (2000). Mutations in the N-terminus of the X-linked retinitis pigmentosa protein RP2 interfere with the normal targeting of the protein to the plasma membrane. Hum. Mol. Genet. *9*, 1919–1926.
35. Cradick, T.J., Qiu, P., Lee, C.M., Fine, E.J., and Bao, G. (2014). COSMID: A Web-based Tool for Identifying and Validating CRISPR/Cas Off-target Sites. Mol. Ther. Nucleic Acids *3*, e214.
36. Benati, D., Miselli, F., Cocchiarella, F., Patrizi, C., Carretero, M., Baldassarri, S., Ammendola, V., Has, C., Colloca, S., Del Rio, M., et al. (2018). CRISPR/Cas9-Mediated In Situ Correction of LAMB3 Gene in Keratinocytes Derived from a Junctional Epidermolysis Bullosa Patient. Mol. Ther. *26*, 2592–2603.

37. Athanasiou, D., Aguila, M., Opefi, C.A., South, K., Bellingham, J., Bevilacqua, D., Munro, P.M., Kanuga, N., Mackenzie, F.E., Dubis, A.M., et al. (2017). Rescue of mutant rhodopsin traffic by metformin-induced AMPK activation accelerates photoreceptor degeneration. Hum. Mol. Genet. *26*, 305–319.
38. Liang, F.Q., Anand, V., Maguire, A.M., and Bennett, J. (2001). Intraocular delivery of recombinant virus. Methods Mol. Med. *47*, 125–139.
39. Tornabene, P., Trapani, I., Minopoli, R., Centrulo, M., Lupo, M., de Simone, S., Tiberi, P., Dell'Aquila, F., Marrocco, E., Iodice, C., et al. (2019). Intein-mediated protein trans-splicing expands adeno-associated virus transfer capacity in the retina. Sci. Transl. Med. *11*, eaav4523.
40. Pinello, L., Canver, M.C., Hoban, M.D., Orkin, S.H., Kohn, D.B., Bauer, D.E., and Yuan, G.C. (2016). Analyzing CRISPR genome-editing experiments with CRISPResso. Nat. Biotechnol. *34*, 695–697.
41. Dunn, K.C., Aotaki-Keen, A.E., Putkey, F.R., and Hjelmeland, L.M. (1996). ARPE-19, a human retinal pigment epithelial cell line with differentiated properties. Exp. Eye Res. *62*, 155–169.
42. Chen, Y., Chen, Y., Jastrzebska, B., Golczak, M., Gulati, S., Tang, H., Seibel, W., Li, X., Jin, H., Han, Y., et al. (2018). A novel small molecule chaperone of rod opsin and its potential therapy for retinal degeneration. Nat. Commun. *9*, 1976.
43. Zhang, Y., Li, H., Min, Y.L., Sanchez-Ortiz, E., Huang, J., Mireault, A.A., Shelton, J.M., Kim, J., Mammen, P.P.A., Bassel-Duby, R., et al. (2020). Enhanced CRISPR-Cas9 correction of Duchenne muscular dystrophy in mice by a self-complementary AAV delivery system. Sci. Adv. *6*, eaay6812.
44. Wang, L., Yang, Y., Breton, C., Bell, P., Li, M., Zhang, J., Che, Y., Saveliev, A., He, Z., White, J., et al. (2020). A mutation-independent CRISPR-Cas9-mediated gene targeting approach to treat a murine model of ornithine transcarbamylase deficiency. Sci. Adv. *6*, eaax5701.
45. Nishiguchi, K.M., Fujita, K., Miya, F., Katayama, S., and Nakazawa, T. (2020). Single AAV-mediated mutation replacement genome editing in limited number of photoreceptors restores vision in mice. Nat. Commun. *11*, 482.
46. Berson, E.L., Rosner, B., Sandberg, M.A., Hayes, K.C., Nicholson, B.W., Weigel-DiFranco, C., and Willett, W. (1993). A randomized trial of vitamin A and vitamin E supplementation for retinitis pigmentosa. Arch. Ophthalmol. *111*, 761–772.
47. Hoffman, D.R., Locke, K.G., Wheaton, D.H., Fish, G.E., Spencer, R., and Birch, D.G. (2004). A randomized, placebo-controlled clinical trial of docosahexaenoic acid supplementation for X-linked retinitis pigmentosa. Am. J. Ophthalmol. *137*, 704–718.
48. Sacchetti, M., Mantelli, F., Merlo, D., and Lambiase, A. (2015). Systematic Review of Randomized Clinical Trials on Safety and Efficacy of Pharmacological and Nonpharmacological Treatments for Retinitis Pigmentosa. J. Ophthalmol. *2015*, 737053.
49. Cehajic-Kapetanovic, J., Xue, K., Martinez-Fernandez de la Camara, C., Nanda, A., Davies, A., Wood, L.J., Salvetti, A.P., Fischer, M.D., Aylward, J.W., Barnard, A.R., et al. (2020). Initial results from a first-in-human gene therapy trial on X-linked retinitis pigmentosa caused by mutations in RPGR. Nat. Med. *26*, 354–359.
50. Garafalo, A.V., Cideciyan, A.V., Héon, E., Sheplock, R., Pearson, A., WeiYang Yu, C., Sumaroka, A., Aguirre, G.D., and Jacobson, S.G. (2020). Progress in treating inherited retinal diseases: Early subretinal gene therapy clinical trials and candidates for future initiatives. Prog. Retin. Eye Res. *77*, 100827.
51. Henrie, A., Hemphill, S.E., Ruiz-Schultz, N., Cushman, B., DiStefano, M.T., Azzariti, D., Harrison, S.M., Rehm, H.L., and Eilbeck, K. (2018). ClinVar Miner: Demonstrating utility of a Web-based tool for viewing and filtering ClinVar data. Hum. Mutat. *39*, 1051–1060.
52. Lewin, A.S., Rossmiller, B., and Mao, H. (2014). Gene augmentation for adRP mutations in RHO. Cold Spring Harb. Perspect. Med. *4*, a017400.
53. Price, B.A., Sandoval, I.M., Chan, F., Nichols, R., Roman-Sanchez, R., Wensel, T.G., and Wilson, J.H. (2012). Rhodopsin gene expression determines rod outer segment size and rod cell resistance to a dominant-negative neurodegeneration mutant. PLoS ONE *7*, e49889.
54. Mao, H., James, T., Jr., Schwein, A., Shabashvili, A.E., Hauswirth, W.W., Gorbatyuk, M.S., and Lewin, A.S. (2011). AAV delivery of wild-type rhodopsin preserves retinal function in a mouse model of autosomal dominant retinitis pigmentosa. Hum. Gene Ther. *22*, 567–575.
55. Tsai, Y.T., Wu, W.H., Lee, T.T., Wu, W.P., Xu, C.L., Park, K.S., Cui, X., Justus, S., Lin, C.S., Jauregui, R., et al. (2018). Clustered Regularly Interspaced Short Palindromic Repeats-Based Genome Surgery for the Treatment of Autosomal Dominant Retinitis Pigmentosa. Ophthalmology *125*, 1421–1430.
56. Apaja, P.M., and Lukacs, G.L. (2014). Protein homeostasis at the plasma membrane. Physiology (Bethesda) *29*, 265–277.
57. Mussolino, C., della Corte, M., Rossi, S., Viola, F., Di Vicino, U., Marrocco, E., Neglia, S., Doria, M., Testa, F., Giovannoni, R., et al. (2011). AAV-mediated photoreceptor transduction of the pig cone-enriched retina. Gene Ther. *18*, 637–645.
58. Maeder, M.L., Stefanidakis, M., Wilson, C.J., Baral, R., Barrera, L.A., Bounoutas, G.S., Bumcrot, D., Chao, H., Ciulla, D.M., DaSilva, J.A., et al. (2019). Development of a gene-editing approach to restore vision loss in Leber congenital amaurosis type 10. Nat. Med. *25*, 229–233.
59. Kim, E., Koo, T., Park, S.W., Kim, D., Kim, K., Cho, H.Y., Song, D.W., Lee, K.J., Jung, M.H., Kim, S., et al. (2017). In vivo genome editing with a small Cas9 orthologue derived from Campylobacter jejuni. Nat. Commun. *8*, 14500.
60. Yu, W., Mookherjee, S., Chaitankar, V., Hiriyanna, S., Kim, J.W., Brooks, M., Ataeijannati, Y., Sun, X., Dong, L., Li, T., et al. (2017). Nrl knockdown by AAV-delivered CRISPR/Cas9 prevents retinal degeneration in mice. Nat. Commun. *8*, 14716.

The American Journal of Human Genetics, Volume *108*

Supplemental Data

Allele-specific editing ameliorates dominant retinitis pigmentosa in a transgenic mouse model

Clarissa Patrizi, Manel Llado, Daniela Benati, Carolina Iodice, Elena Marrocco, Rosellina Guarascio, Enrico M. Surace, Michael E. Cheetham, Alberto Auricchio, and Alessandra Recchia

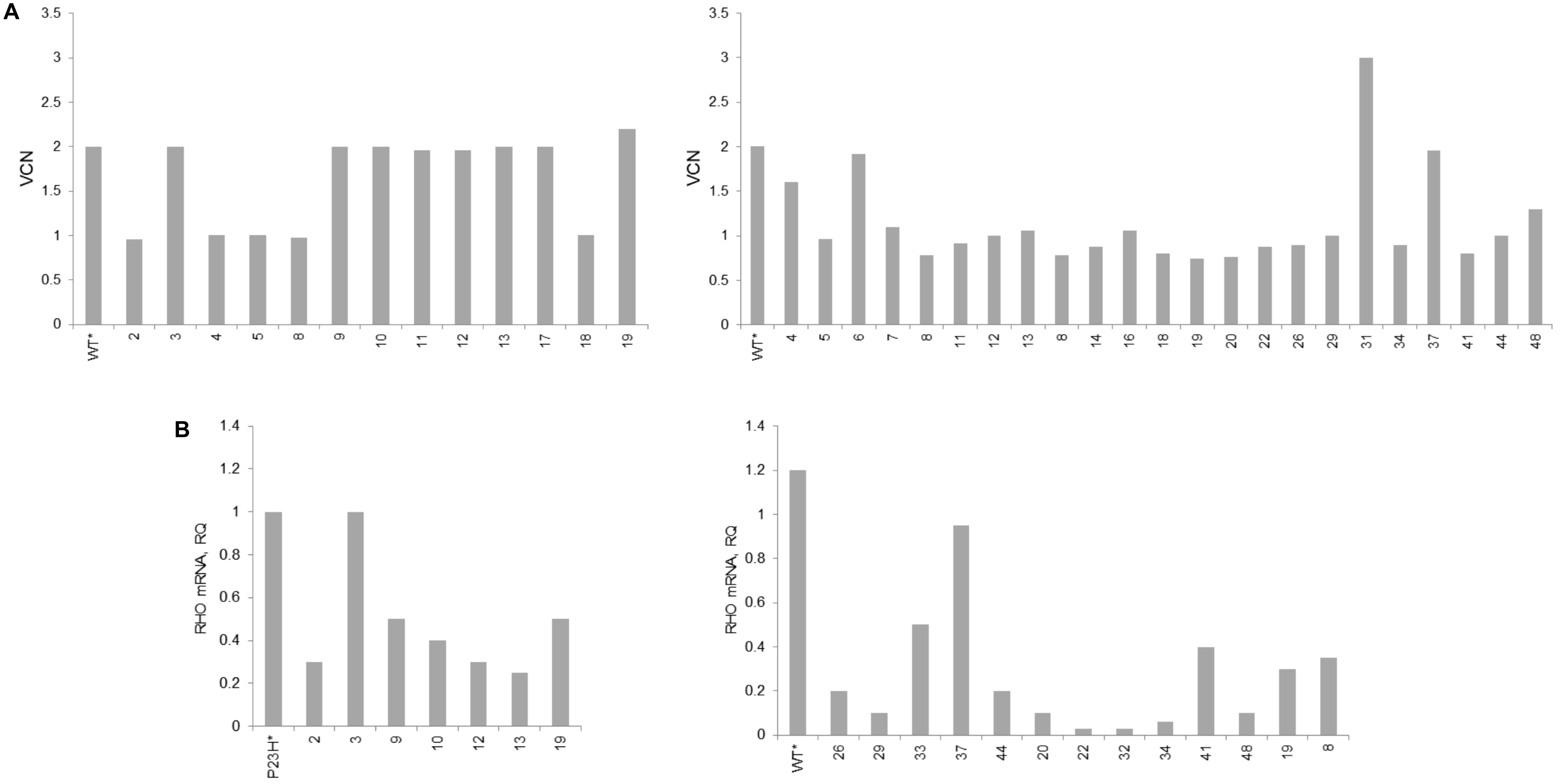

Figure S1. Engineering HeLa cells to express WT or P347S *RHO. A*) qPCR on P347S (left panel) and WT (right panel) *RHO* HeLa clones to determine vector copy number (VCN) of PGK-driven expression cassettes carrying WT or P347S *RHO* cDNA and part of the 3'UTR region. To select clones hosting 2 copies of the WT or P347S transgene, a WT* clone isolated and used in Latella *et al.*[29] was included as reference. **B)** Quantitative expression of *RHO* mRNA in P347S (left panel) or WT *RHO* (right panel) clones compared to a P23H* and WT* clones isolated and used in Latella *et al*.[29] . Clone #3 belonging to P347S RHO and clone #37 belonging to WT RHO were selected for *in vitro* editing experiments performed in this study.

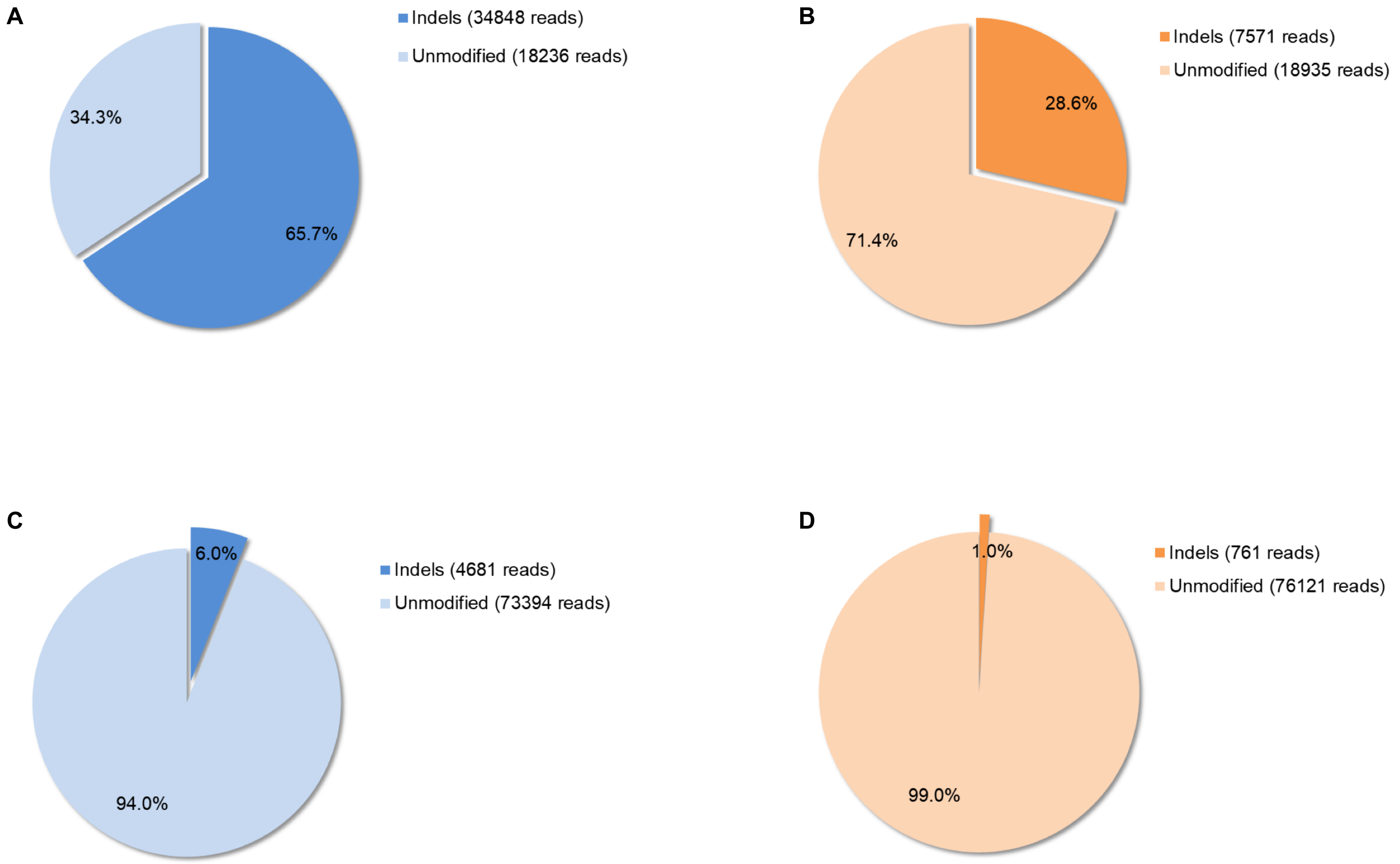

Figure S2. Allele-specific editing of P347S RHO transgene *in vitro*. P347S HeLa clone transfected with SpCas9_gRNA1 (**A**) or VQRHF1-SpCas9_gRNA5 (**B**) underwent NGS analysis tailored to the target site. NGS sequence reads were analyzed by CRISPResso software which retrieved a frequency of reads bearing indels or unmodified. Pie charts of the results from a representative experiment are reported. **C, D)** Pie charts of a representative experiment showing frequency and amount of reads carrying indels or unmodified reads scored in WT *RHO* HeLa clone transfected with SpCas9_gRNA1 (**C**) or -VQRHF1-SpCas9_gRNA5 (**D**).

A

	Potential splice site modified	Unmodified
THRA	0%	100%
ROR1	0%	100%
RBSN	0%	100%
EPSILS1	0%	100%

B

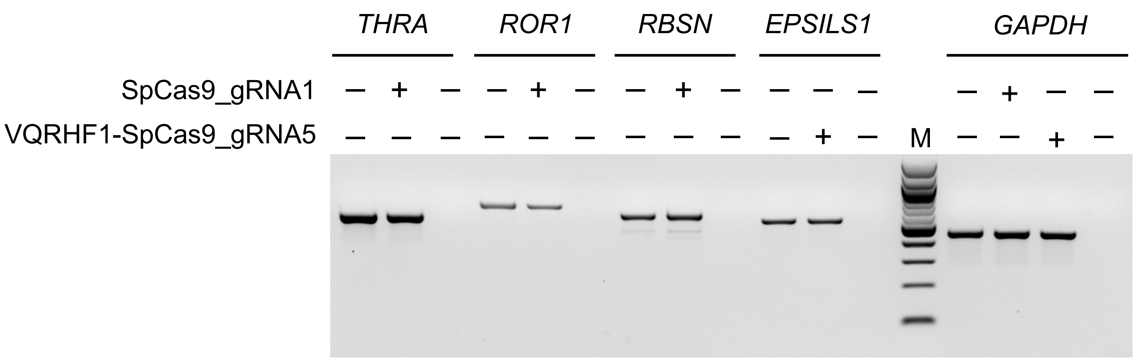

C

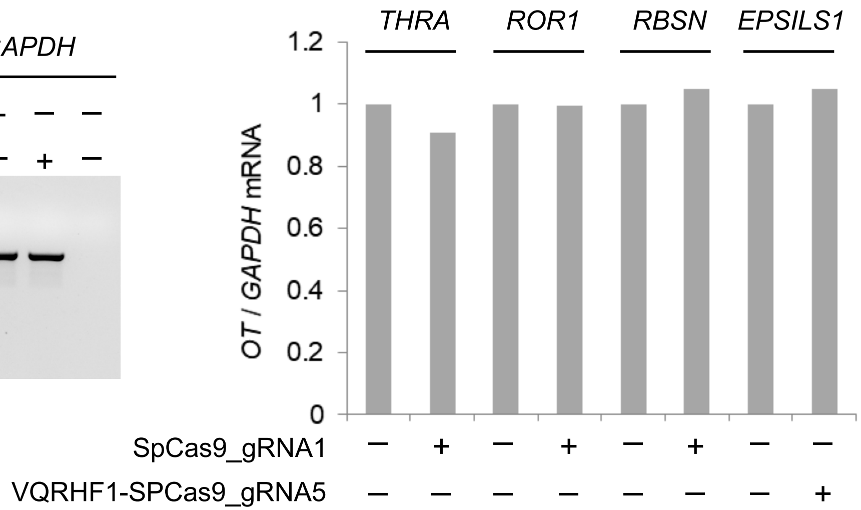

Figure S3. Unmodified expression of off-target genes upon CRISPR-mediated editing. A) CRISPResso analysis of potential splice sites in *THRA*, *ROR1*, *RBSN* and *EPSILS1* modified upon editing induced by SpCas9_gRNA1 or VQRHF1-SpCas9_gRNA5. **B)** RT-PCR analysis for the expression of *THRA*, *ROR1*, *RBSN* and *EPSILS1* in human RPE cells transfected or not with effector plasmids for the expression of SpCas9_gRNA1 or VQRHF1-SpCas9_gRNA5. RT-PCR analysis of *GAPDH* expression was evaluated as control. M, 100 bp molecular weight marker. **C)** Densitometric quantification of *THRA*, *ROR1*, *RBSN* and *EPSILS1* mRNA (OT), normalised to *GAPDH* in RPE cells transfected or not as indicated in panel (B).

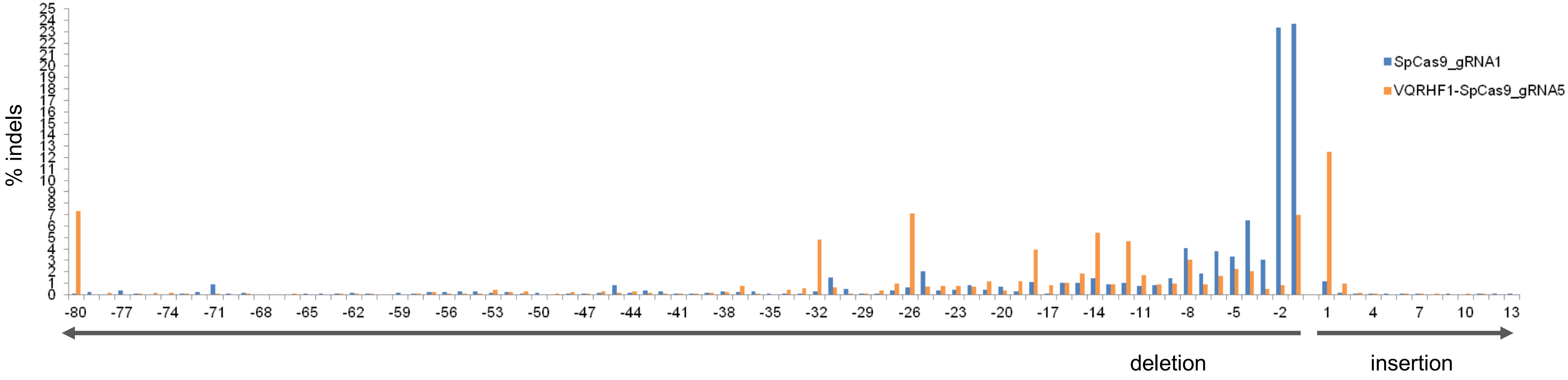

Figure S4. Distribution of indels in CRISPR-edited P347S *RHO* HeLa clone. Profile of indels scored in P347S *RHO* HeLa clone transfected with SpCas9_gRNA1 or VQRHF1-SpCas9_gRNA5. Deletions are represented by minus numbers whereas plus numbers represent insertions. Sequences without indels (value = 0) are omitted from the chart.

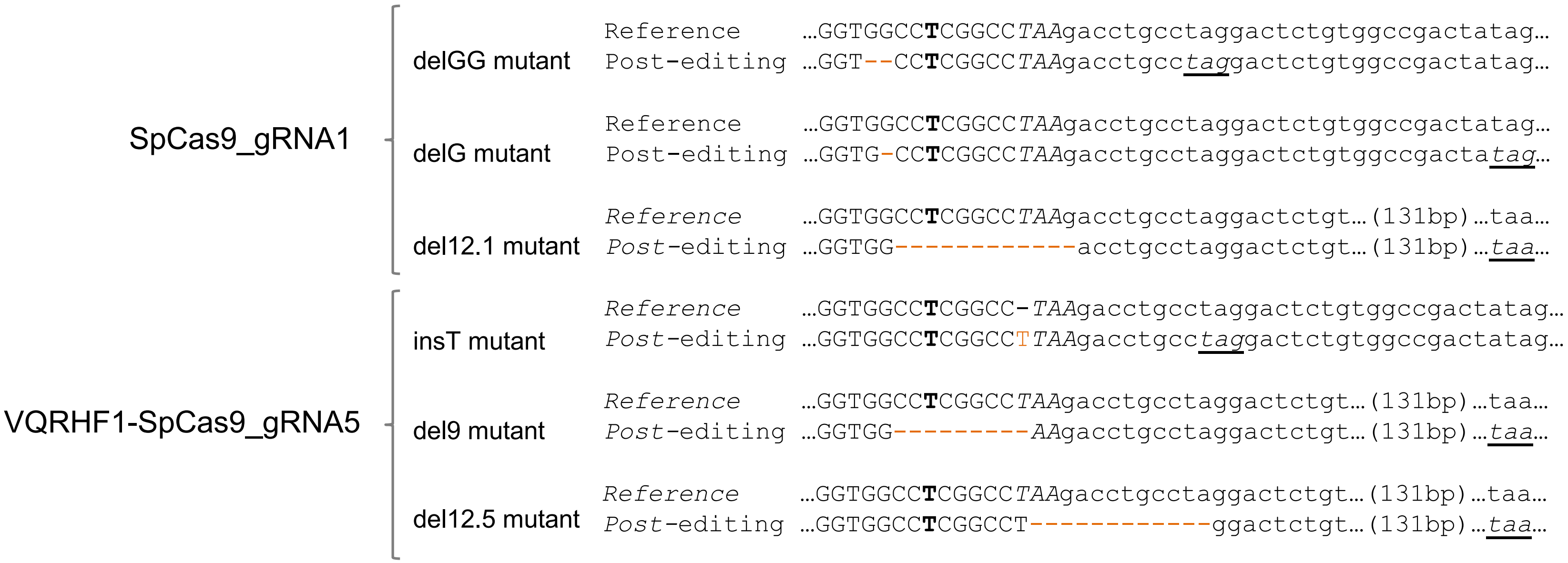

Figure S5. Most frequent RHO mutants generated upon CRISPR-mediated editing of P347S *RHO* mutation *in vitro*. Nucleotide sequence of the six most frequent mutants identified by CRISPResso upon editing mediated by SpCas9_gRNA1 or VQRHF1-SpCas9_gRNA5. The C to T conversion resulting in P347S mutation is highlighted in bold. Nucleotides belonging to the 3'-UTR are in italic. Dash indicates a deleted nucleotide, while insertion is shown in orange. New stop codons downstream the canonical one (capital letters in italics) are underlined.

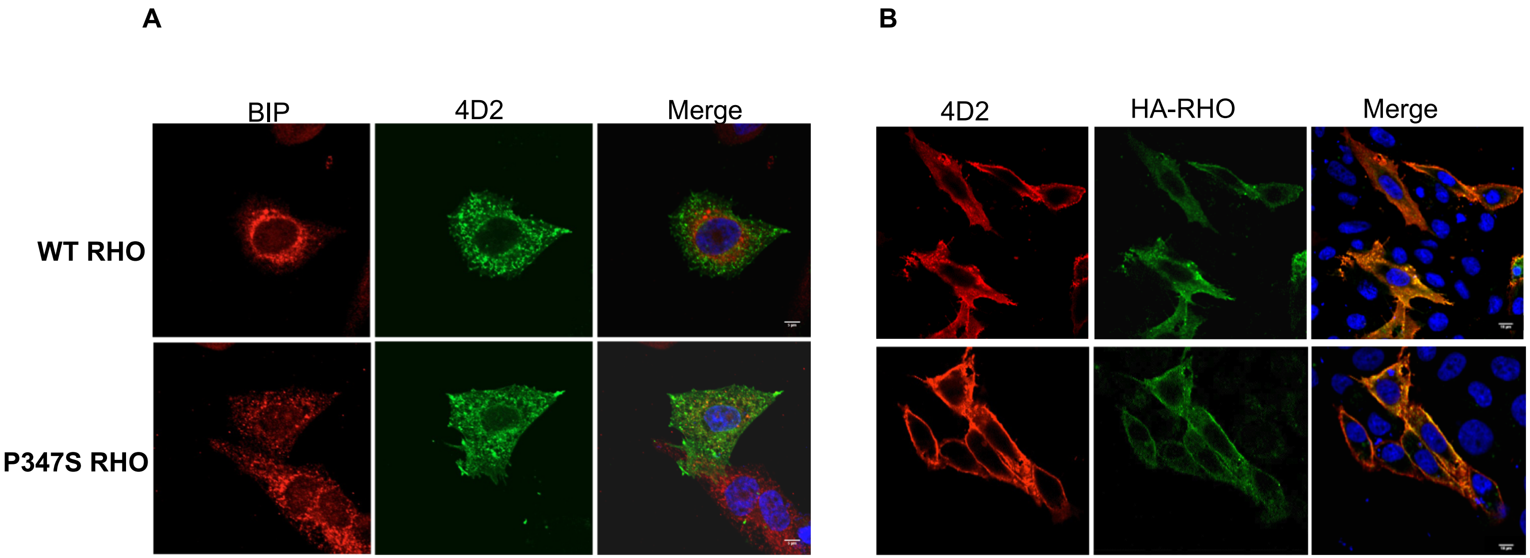

Figure S6. Localization of WT and P347S RHO protein in CHO cells. Immunofluorescence analyses of WT and P347S RHO protein performed in CHO cells transfected with plasmids expressing RHO fused at the N-terminal region to a human influenza hemagglutinin (HA) tag. **A**) Permeabilized cells were stained with anti-BIP and 4D2 antibodies and scale bar is 5 µm. **B**) Non-permeabilized cells were stained with 4D2 and anti-HA antibodies and scale bar is 10 µm. The panels generated merging the signals includes also a DAPI staining of the nuclei.

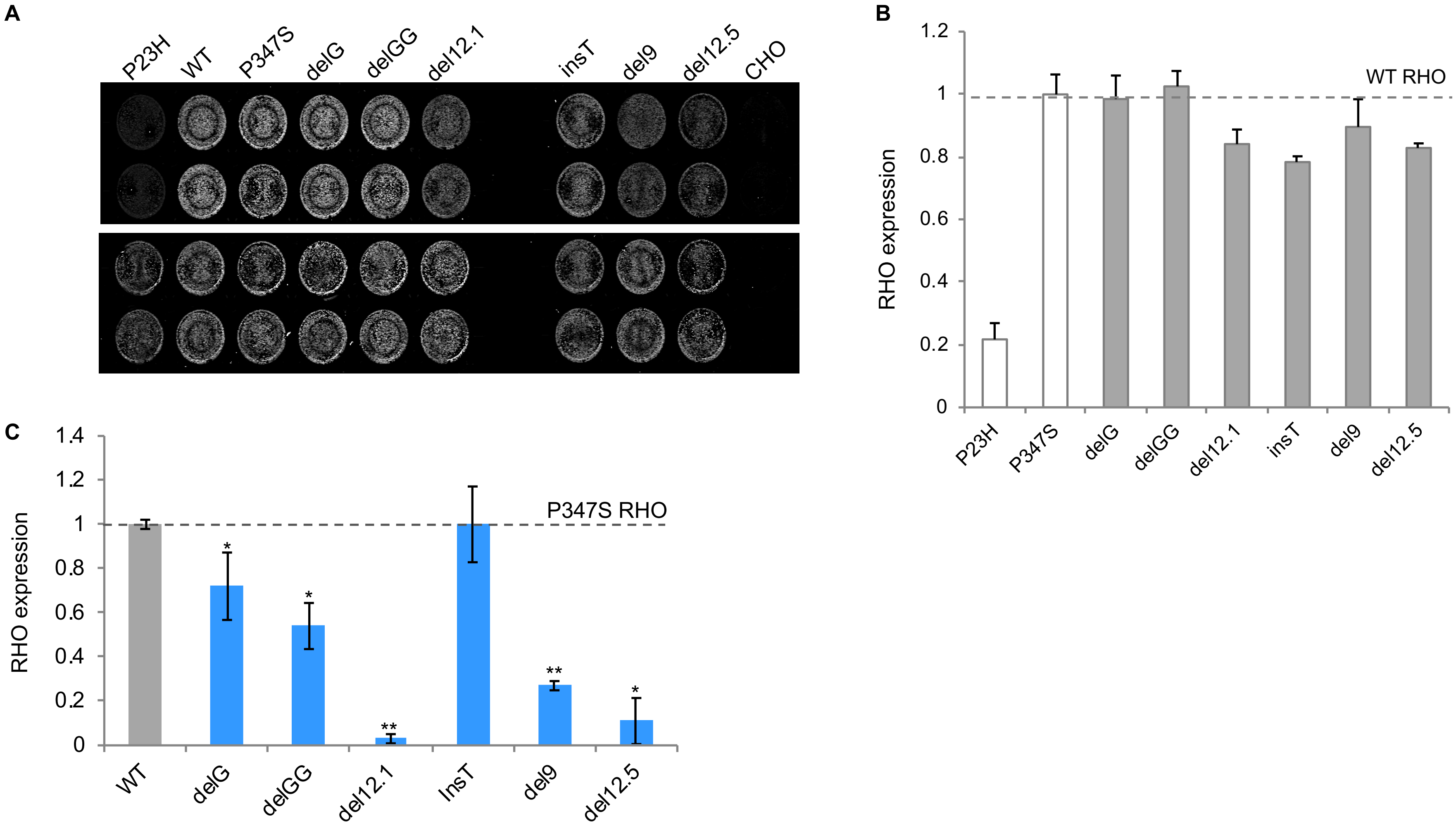

Figure S7. Localization and degradation of RHO mutants generated upon editing of P347S RHO mutation *in vitro*. A) In-Cell Western analysis of non-permeabilized (top panel) and permeabilized (bottom panel) CHO cells transfected with RHO mutant plasmids and stained with anti-HA antibody. As control P23H, WT and P347S RHO expression plasmids were transfected. Mock transfected cells (CHO) are included in the assay. **B)** Graphic representation of the ratio between rhodopsin localized to the plasma membrane over the total rhodopsin detected in "In Cell Western" analysis. The experiment was performed in duplicate and presented as mean ± SEM. **C)** Densitometric quantification of RHO protein level detected by western blot (Figure 2B) in CHO cells transfected with plasmids for the expression of RHO P347S, WT or mutants. The experiment was performed in duplicate and presented as mean ± SEM. * p-value < 0.05, ** p-value < 0.01.

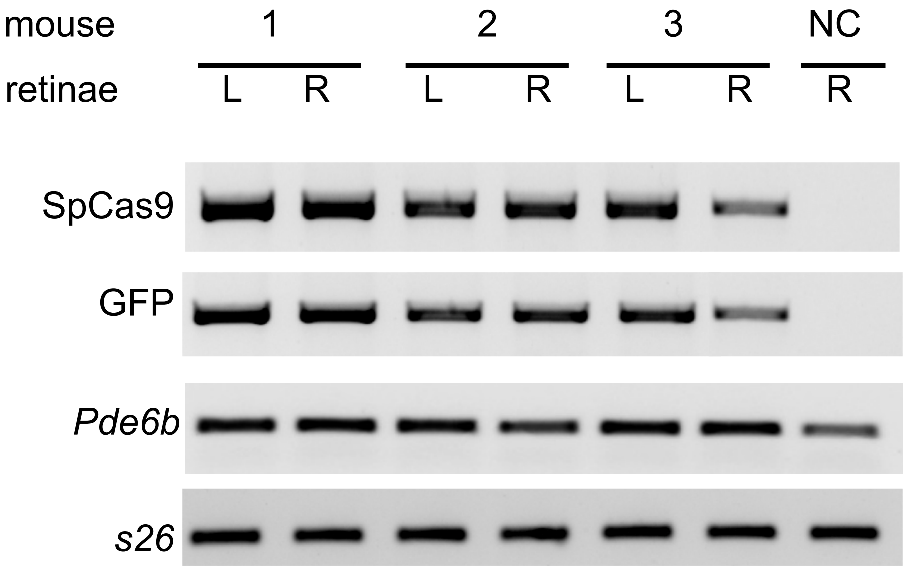

Figure S8. Expression of AAV-CRISPR effector vectors in P347S transgenic mice. RT-PCR analysis for the expression of SpCas9 (WT, mouse #1 and #2 L and R or VQRHF1, mouse #3 L and R) and GFP in retinae of P347S transgenic mice treated with effector or control AAV2/8 vectors. The expression of the rod photoreceptor gene *Pde6b* and the ribosomal *s26* RNA was used for normalization. L and R indicate left and right eye, respectively. NC, negative control, not injected retina.

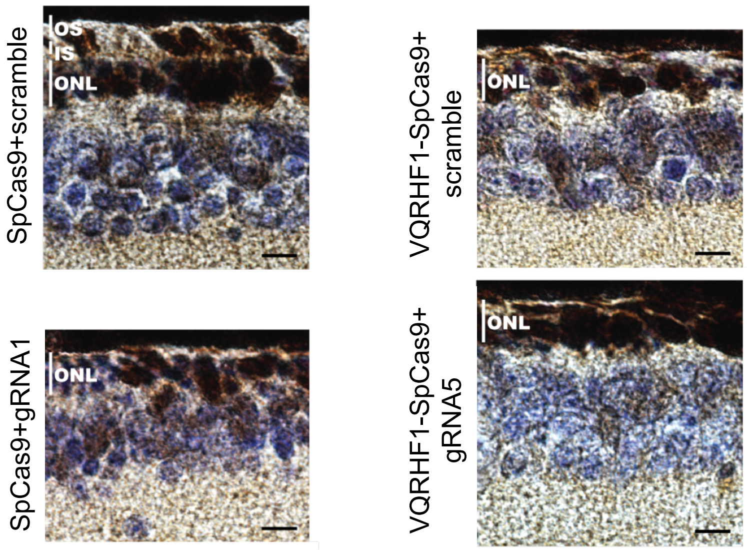

Figure S9. GFP expression in P347S transgenic mice treated with CRISPR-AAVs. Immunohistochemistry of retinal sections derived from mice injected with SpCas9+gRNA1, VQRHF1-SpCas9+gRNA5 or SpCas9+scramble vectors. Anti-GFP antibody stained the AAV carrying the gRNAs. Outer segment (OS), inner segment (IS) and outer nuclear layer (ONL) are indicated. Scale bar corresponding to 10 μm is shown.
